# Unprecedented tunability of riboswitch structure and regulatory function by sub-millimolar variations in physiological Mg^2+^

**DOI:** 10.1093/nar/gkz316

**Published:** 2019-05-02

**Authors:** Kaley McCluskey, Julien Boudreault, Patrick St-Pierre, Cibran Perez-Gonzalez, Adrien Chauvier, Adrien Rizzi, Pascale B Beauregard, Daniel A Lafontaine, J Carlos Penedo

**Affiliations:** 1 SUPA School of Physics and Astronomy, University of St. Andrews, Scotland KY16 9SS, UK; 2 Département de Biologie, Université de Sherbrooke, Québec, Canada J1K 2R1; 3 Centre SÈVE, Département de Biologie, Faculté des Sciences, Université de Sherbrooke, Sherbrooke, Canada; 4 Département de Chimie, Faculté des Sciences, Université de Sherbrooke, Sherbrooke, Canada; 5 Biomedical Sciences Research Complex, School of Biology, University of St. Andrews, Scotland KY16 9ST, UK

## Abstract

Riboswitches are *cis*-acting regulatory RNA biosensors that rival the efficiency of those found in proteins. At the heart of their regulatory function is the formation of a highly specific aptamer–ligand complex. Understanding how these RNAs recognize the ligand to regulate gene expression at physiological concentrations of Mg^2+^ ions and ligand is critical given their broad impact on bacterial gene expression and their potential as antibiotic targets. In this work, we used single-molecule FRET and biochemical techniques to demonstrate that Mg^2+^ ions act as fine-tuning elements of the amino acid-sensing *lysC* aptamer's ligand-free structure in the mesophile *Bacillus subtilis*. Mg^2+^ interactions with the aptamer produce encounter complexes with strikingly different sensitivities to the ligand in different, yet equally accessible, physiological ionic conditions. Our results demonstrate that the aptamer adapts its structure and folding landscape on a Mg^2+^-tunable scale to efficiently respond to changes in intracellular lysine of more than two orders of magnitude. The remarkable tunability of the *lysC* aptamer by sub-millimolar variations in the physiological concentration of Mg^2+^ ions suggests that some single-aptamer riboswitches have exploited the coupling of cellular levels of ligand and divalent metal ions to tightly control gene expression.

## INTRODUCTION

**Figure 1.**
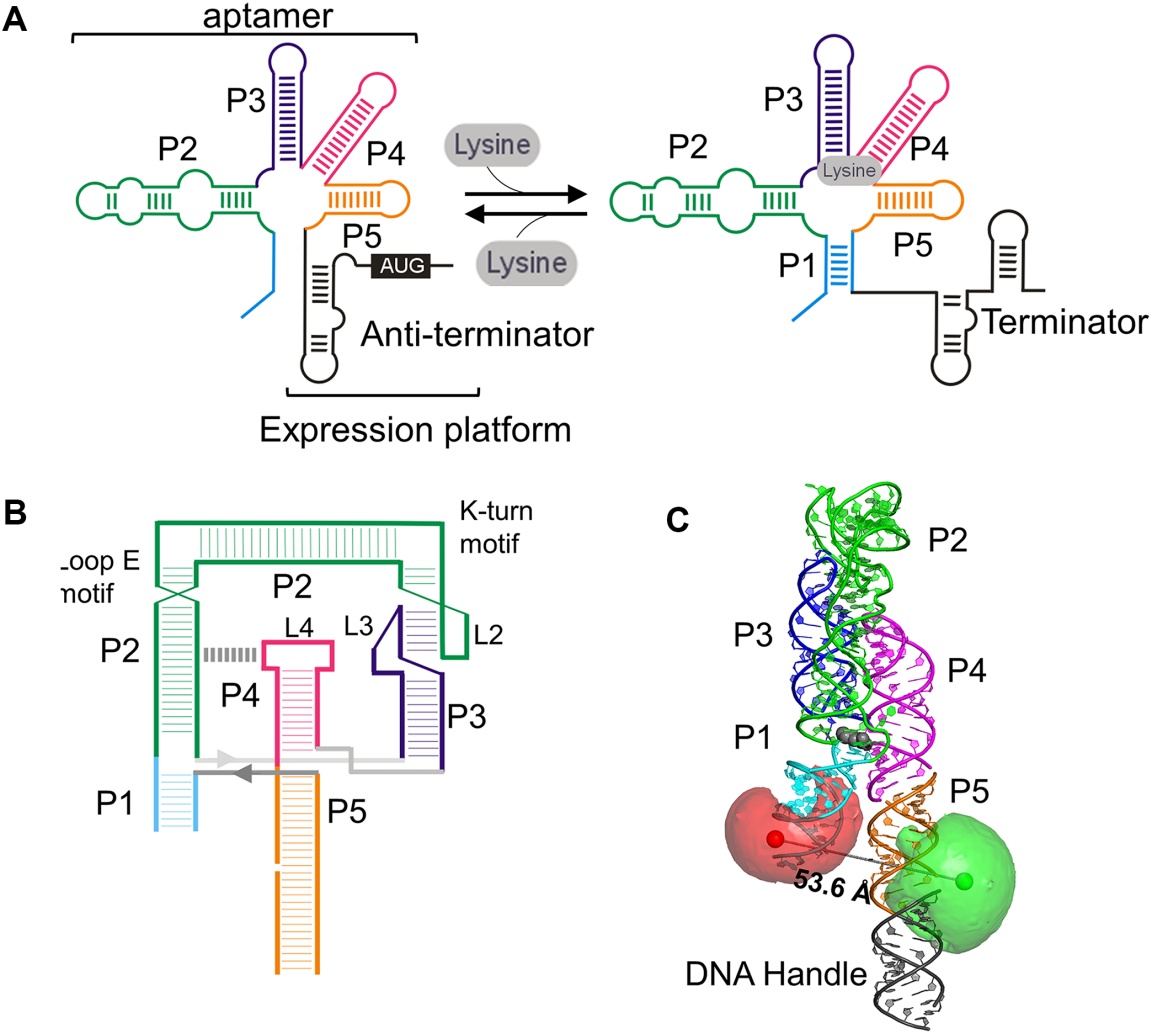
Structural components and regulatory mechanism of the lysine riboswitch aptamer. (**A**) Secondary structure of the *lysC* riboswitch showing the aptamer domain, comprising stems P1 to P5, and the expression platform. Predicted changes in the expression platform induced by lysine binding are also shown. In the absence of ligand an anti-terminator stem forms, allowing transcription readthrough. Upon ligand binding, the expression platform forms a terminator stem that stops transcription. (**B**) Tertiary contacts in the lysine-bound aptamer as observed in the crystal structure. In addition to the P1–P2 and P4–P5 helical stacks, the L2–L3 and P2–L4 interactions assisted by k-turn and loop E motifs, respectively, are also shown. The P5 stem of the experimental construct, shown here, has been extended to include a biotinylated DNA portion for surface immobilization. (**C**) X-ray crystal structure of the ligand-bound lysine aptamer (PDB accession code 3DIL) (20). The positions of the FRET donor Cy3 (green) and the FRET acceptor Cy5 (red) used in this study are shown. Mean dye positions were modelled using the accessible volume (AV) approach (Supplementary Methods). The ligand is shown in grey, and the line between the donor and acceptor spheres represents the predicted mean distance.

Riboswitches are noncoding mRNA sequences usually found in the 5′ untranslated regions of many genes involved in metabolite biosynthesis or transport, which they regulate by binding specific, related metabolites (1,2). Riboswitch architecture includes an aptamer domain that acts as the metabolite-sensing element and a downstream expression platform that interacts with the transcription or translation machinery (3–5). A cascade of local and long-range conformational changes is initiated by ligand binding to the aptamer and transmitted to the expression platform (6), biasing its structure towards one of two competing conformers that control the activation or repression of the downstream gene (Figure 1A). Riboswitches have been shown to regulate gene expression through different mechanisms including transcription termination, translation inhibition and, in some eukaryotes, by exposing an alternative mRNA splicing site (1,5,7).

To date, nearly 40 classes of riboswitches have been discovered (8), among them, three amino acid-sensing riboswitch classes that recognize lysine (8,9), glycine (10) or l-glutamine (11). Lysine riboswitches typically regulate the expression of lysine-specific enzymes in the diaminopimelate (DAP) pathway for lysine, methionine and threonine biosynthesis (12). However, despite the lysine riboswitch's abundance (13), biological relevance (14), and proven potential as antibiotic target (15) and in bioengineering (16), its regulatory mechanism remains poorly understood.

The *lysC* aptamer from *Bacillus subtilis* comprises five helical stem-loops, labelled P1-P5 in Figure 1A, organised around a core domain (9,17–20) (Figure 1B and c). In the presence of lysine, P1 is stabilized by close association with P5, precluding the formation of an anti-terminator stem and allowing a terminator to form and eject the transcription complex (19,20). Recent studies have revealed the structural motifs and residues responsible for its ligand specificity (21,22). However, many questions about its structural dynamics remain unexplored, including how its folding landscape changes as a function of ionic environment and the ligand; and whether the ligand itself participates in folding. Small-angle X-ray scattering (SAXS) (23) and crystallographic data suggest very little difference between ligand-bound and ligand-free structures (9,20). The crystallized aptamers were from the thermophile *Thermotoga maritima*, which is extremely stable and has a 30-fold greater affinity for lysine than the aptamer from mesophilic *B. subtilis* (20). Biochemical data on the *B. subtilis* aptamer, on the other hand, provide evidence for a global rearrangement of the aptamer upon ligand binding (9,17).

In this work, we have quantified the *in vivo* concentration of Mg^2+^ ions in *B. subtilis* and used single-molecule FRET (24–26) to explore the conformational dynamics and ligand-binding response of the *B. subtilis lysC* aptamer within the *in vivo* range of Mg^2+^ concentrations. We reveal a remarkable tunability of the *lysC* folding pathway in response to subtle changes in the physiological concentration of Mg^2+^ ions. We demonstrate that sub-millimolar variations in Mg^2+^ impact aptamer function, alter the structure of the aptamer–ligand encounter complex, and change the ligand-binding affinity by two orders of magnitude. The extreme sensitivity of the *lysC* aptamer suggests it acts as a dual-input sensor that relies on Mg^2+^ and lysine levels to ensure tight control over gene expression. Our results imply that cellular levels of Mg^2+^ ions not only participate in RNA compaction and stabilization but could also act as modulators of gene expression. Importantly, the *lysC* aptamer does not fold into its global *holo* state in the absence of lysine, regardless of the Mg^2+^ concentration. This is unusual for the riboswitch aptamers that have been studied with single-molecule techniques to date, which often adopt a ligand-free folded conformation that may be only kinetically distinguishable from the ligand-bound state.

## MATERIALS AND METHODS

### Labelling and purification of RNA oligonucleotides

The experimental constructs for the wild type and variant lysine aptamers incorporate two RNA strands and a biotin-carrying DNA tether (see Supplementary Methods for details). The longest strand was generated by *in vitro* transcription, whilst the other two were synthetic (IDT Inc., USA). The sequences and a schematic of the construct are shown in Supplementary Table S1 and Supplementary Figure S3. Fluorophores were incorporated at the appropriate location either during solid-phase chemistry or post-synthesis using succinimide ester derivatives of Cy3 and Cy5 and following the protocol provided by the dye manufacturer (GE Lifesciences, USA) as described in the Supplementary Methods. Purification of single-strand RNA sequences and hybridized constructs was carried out using polyacrylamide gel electrophoresis, also as described in the Supplementary Methods.

### Single-round *in vitro* transcription

DNA templates were prepared by PCR using the *B. subtilis glyQs* promoter followed by the *B. subtilis lysC* riboswitch aptamer domain fused to a 95 nt sequence downstream of the expression platform. To allow transcription to be initiated by the ApC dinucleotide, the promoter region ends with an adenine and was fused to the C17 position of the riboswitch sequence. The truncated product terminates at position 269, whereas for the full-length product the RNAP continues 95 nt further. Reactions were analysed by gel electrophoresis as previously described (17) and performed at least three times.

### Intracellular Mg^2+^ measurement

*Bacillus subtilis* strain 168 was grown in a Spizizen medium at 37°C (150 rpm) for 4 h (27). Every hour, an aliquot of the culture was collected, and cells were pelleted by centrifugation (Beckman Coulter_TM_ J-25I with JLA 16,250 rotor; 6500g, 20°C, 7 min). The cells were then suspended in 4% paraformaldehyde in PBS and incubated 7 min at room temperature for fixation. Cells were pelleted by centrifugation and washed with an oxalate/EDTA solution (0.1 M/0.05 M) at room temperature for 7 min to remove external metals. The pellet was then suspended in 10 ml sterile 0.5 M NaCl. From this 10 ml, 2 ml were coloured with SYTO9 and used for cell volume calculation. Cellular volume was obtained using fluorescent microscopy pictures of *B. subtilis* taken with the fluorescent dye and it was calculated as a cylinder and two half-spheres using maximum Feret diameter (length) and minimal Feret diameter (width) evaluated by image analysis (CellProfiler 3). Images were taken using a Zeiss Axio Observer Z1 microscope. The other 8 ml were washed again with oxalate/EDTA and pelleted by centrifugation (6500g, 20°C, 7 min). These cells were digested on an SCP science Digiprep Jr with 600 μl of nitric acid (trace metal grade, Fisher Chemical) at 65°C for 45 min. The SCP science Digiprep Jr is a 24-position Teflon-coated block digestion system that can reach 180°C with 1.0°C uniformity across the surface. After digestion, each tube was filled with 10 ml Milli-Q water. Samples were analyzed for phosphorus and metal content on an inductively-coupled-plasma mass spectrometer (ICP-MS; Thermo Scientific, XSeries2) as previously described (28). ICP-MS allows detection and quantification of metals at low concentration (ppt). Analysis is achieved by ionizing an acid-digested sample with a plasma torch of argon. Ions are then extracted through a series of cones into a quadrupole and separated by their mass/charge ratio before reaching the detector. Phosphorus content was used as a proxy to evaluate cell number.

### Single-molecule FRET

Single-molecule FRET experiments were carried out using a Total Internal Reflection microscope that has been described elsewhere (29). The experimental buffer contained 50 mM Tris at pH 7.8, 100 mM K^+^ unless otherwise noted, and Mg^2+^ and l-lysine as indicated. Trolox was used as triplet state quencher (30) and PCA-PCD as the oxygen scavenging system (31). The temporal resolution of the single-molecule movies was 50 ms/frame except to capture the slow dynamics in Figure 5B, where 200 ms/frame was used. FRET was calculated from the raw donor and acceptor intensity traces using ${E_{{\rm app}}} = {{{I_{\rm A}}} \mathord{/ {\vphantom {{{I_A}} {( {{I_{\rm D}} + \alpha {I_{\rm A}}} )}}} } {( {{I_{\rm D}} + \alpha {I_{\rm A}}} )}}$ where α = 0.88 accounts for 12% leakage into the acceptor detection channel (32). When constructing FRET population histograms, the first ten frames of each movie were averaged to produce a ‘average’ value for that movie. Between 1100 and 1500 representative FRET values, and 35–450 structural state dwell times in Figure 5B, were used to construct each distribution in this work.

## RESULTS

### Measurement of the *in vivo* Mg^2+^ concentration in *B. subtilis*

Previous work on the lysine riboswitch has addressed aptamers from multiple organisms, both thermophilic (20,33) and mesophilic (12,23,34), in a wide variety of physiological and non-physiological cationic environments. Recent studies on the *Escherichia coli* metalome suggest free concentrations of Mg^2+^ ions between 1.5 and 3 mM, whereas the total Mg^2+^ concentration, mostly interacting with nucleic acids, proteins and other metabolites, can reach values of ∼ 50 mM (35). Given the lack of data regarding the intracellular concentration of Mg^2+^ ions in *B. subtilis*, we determine the Mg^2+^ concentration range in growth-phase *B. subtilis*, relevant to *lysC* riboswitch function *in vivo*, using inductively-coupled plasma mass spectrometry (ICP-MS).

*Bacillus subtilis* strain 168 was grown in a Spizizen (27) medium at 37°C and 150 rpm, including 1.6 mM final Mg^2+^ concentration provided as MgSO_4_.7H_2_O. Maximum and minimum values of intracellular Mg^2+^ were calculated each hour for 4 h and nine biological replicates. The maximum and minimum Mg^2+^ values were calculated using the maximal and minimal cell volumes observed, respectively (Supplementary Figure S1). The intracellular Mg^2+^ concentrations exhibited very little variation during the growth phase. The minimum average values ranged from 0.8 ± 0.2 to 1.1 ± 0.2 mM, and the maximum average values from 2.6 ± 0.6 mM to 3.7 ± 0.2 mM. These values are similar to those reported for the free Mg^2+^ concentration in *Salmonella enterica* cells either immersed in a Mg^2+^-free medium ([Mg^2+^]_free_ ∼ 0.9 mM) or in 10 mM extracellular Mg^2+^ concentration ([Mg^2+^]_free_ ∼ 1.6 mM) (36,37).

### Transcriptional regulation by *lysC* riboswitches at physiological Mg^2+^ concentrations

Next, we used a single-round *in vitro* transcription assay (12,17) to characterize riboswitch regulatory function within the determined physiological range of Mg^2+^ ions (Supplementary Figure S2A and B). In the absence of lysine, a similar transcription termination baseline of ∼25% was observed in 1, 2 and 10 mM Mg^2+^. However, when a saturating concentration of lysine ligand (5 mM) was added to the transcription buffer, termination efficiency reached a value of 75% in 2 mM Mg^2+^, which decreased to 51% in 1 mM Mg^2+^ (Supplementary Figure S2C). A further increase in the concentration of Mg^2+^ to 10 mM only slightly increased the termination efficiency to a value of 82%. Transcription termination levels of ∼30% in the absence of ligand and maximal termination efficiencies of ∼80% at saturating ligand concentrations are typical for many riboswitches, including the lysine riboswitch (21).

The lysine concentration required to induce a 50% transcription termination (T_50_) decreased by half when the Mg^2+^ concentration increased from 1 to 2 mM, from 129 ± 15 μM to 61 ± 7 μM. However, T_50_ only decreased by an additional 15%, to 52 ± 7 μM, in 10 mM Mg^2+^ (Supplementary Figure S2c). A 50% variation in T_50_ in the 1–2 mM range suggests that *lysC* regulatory function can be strongly modulated by sub-millimolar variations in the physiological concentration of Mg^2+^ ions.

### Influence of Mg^2+^ ions on *lysC* conformation

We wanted to determine the conformational response of the *lysC* aptamer to small changes in Mg^2+^ levels. Therefore, we began by characterizing the structure and dynamics of the ligand-free state as a function of the Mg^2+^ concentration using smFRET in a total-internal reflection (TIR) microscope (38–40). We assembled our experimental *lysC* aptamer from three oligonucleotide strands (Supplementary Figure S3 and Supplementary Table S1). The Cy5 acceptor was incorporated internally in the P1 stem, and the Cy3 donor on a P5 stem that was extended by 15 bp to hybridize a biotinylated DNA strand for surface attachment (Figure 1B and C and Supplementary Figure S3). We have chosen to monitor the P1–P5 distance based on chemical probing and previous FRET data that suggested a significant structural re-organization of these stems on ligand binding (34). We obtained a molecular model of the doubly labelled aptamer using the X-ray structure of the ligand-bound state (PDB: 3DIL) (20) and the mean dye positions calculated using the accessible volume (AV) approach (41) (see Supplementary Methods) (Figure 1C). The modelled Cy3–Cy5 distance was ∼53.6 Å. Assuming a Förster distance of 60 Å we expected an apparent FRET efficiency (*E*_app_) of ∼0.66 for the ligand-bound state.

**Figure 2.**
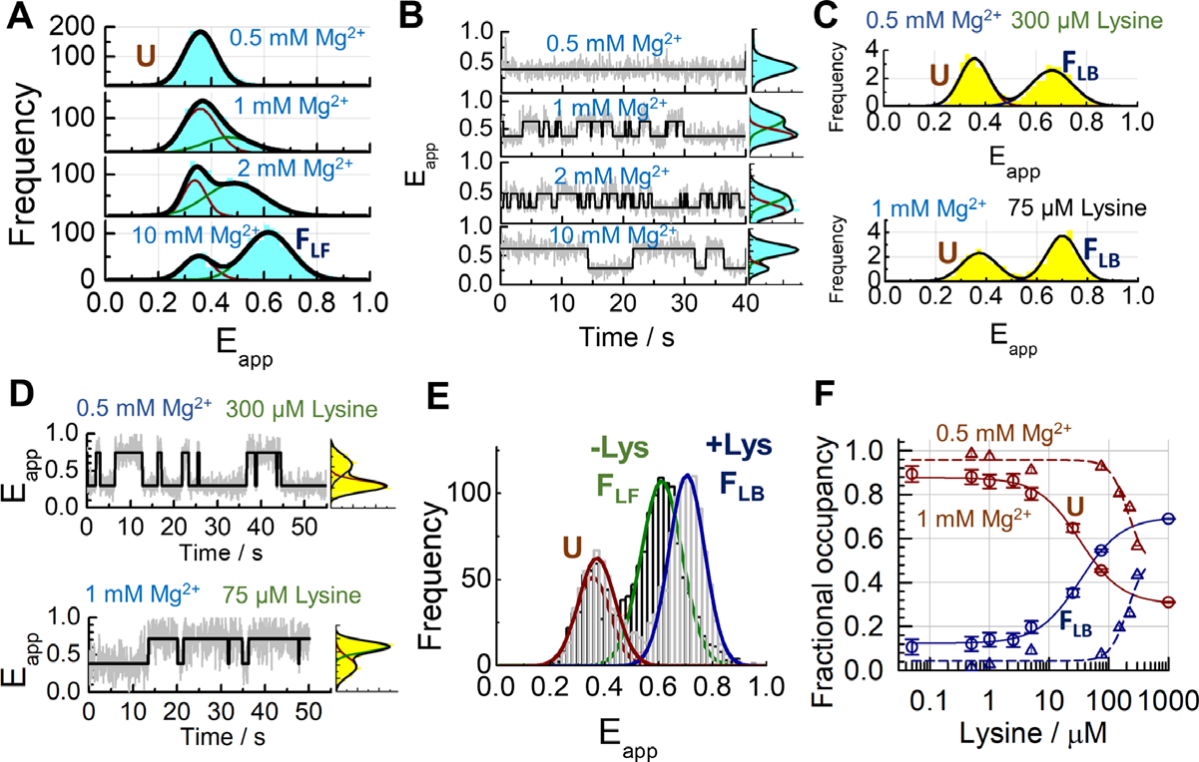
Ligand-binding to unstructured aptamers promotes direct folding into the native state. Single-molecule FRET histograms (**A**) and corresponding FRET trajectories (**B**) obtained at the indicated concentrations of Mg^2+^ ions. (**C**) Comparison of single-molecule FRET histograms obtained in a background of 100 mM K^+^, 0.5 mM Mg^2+^ and 300 μM lysine ligand (top), and in a background of 100 mM K^+^, 1 mM Mg^2+^ and 75 μM lysine (bottom). (**D**) Representative single-molecule FRET trajectories obtained in the same conditions as (C). The solid line represents the idealized FRET trajectory obtained using Hidden Markov modelling. (**E**) Unfolded (**U**, dark red), ligand-free folded (**F_LF_**, green) and ligand-bound folded (**F_LB_**, blue) states have distinct FRET efficiencies. Comparison of single-molecule FRET histograms obtained in the absence (dark grey) and presence of lysine (light grey) are shown. The single-molecule histogram in the absence of lysine with a predominant **F**_**LF**_ state corresponds to that shown at 10 mM Mg^2+^ in panel a. The single-molecule histogram with a predominant F_LB_ state corresponds to that shown in panel C. (**F**) Relative contributions of the **U** (dark red) and **F_LB_** (dark blue) states as a function of lysine concentration obtained in a background of 100 mM K^+^ and 0.5 mM (Δ) or 1 mM (o) Mg^2+^ ions. The solid lines represent the result of a global fit of the contributions of the **U** and **F_LB_** states to a Hill model as a function of lysine concentration (Supplementary Methods).

At low concentrations of monovalent ions (Supplementary Figure S4A) or in the presence of EDTA (Supplementary Figure S5A), the single-molecule FRET histograms showed a single Gaussian peak centered at *E*_app_ ∼ 0.32 ± 0.01(mean ± s.d.), and the smFRET trajectories of individual aptamers remained constant until photobleaching occurred (Supplementary Figures S4B and S5B). We designated this state the unfolded conformation, **U**. As we increased the concentration of Mg^2+^ in a background of 100 mM K^+^, a decrease in the relative contribution of the low-FRET peak and a concomitant increase in the contribution of a higher-FRET peak were observed (Figure 2A). In 10 mM Mg^2+^ (Figure 2A, bottom panel), 70% of the population has shifted into a state centered at *E*_app_ = 0.6 ± 0.01, which we designated the ligand-free folded state, **F_LF_**. A representative single-molecule trace for each Mg^2+^ concentration investigated is shown in Figure 2B. As the Mg^2+^ concentration increases, the dwell time in **U** decreases while the dwell time in the **F_LF_** state increases.

Using the average FRET efficiency obtained from the single-molecule histograms, we fitted the Mg^2+^ binding isotherm to a Hill equation (Supplementary Figure S6A). We obtained a dissociation constant of 1.8 ± 0.3 mM and a Hill coefficient of 2.6 ± 0.4. When these experiments were repeated in the absence of K^+^ ions, the smFRET histograms and trajectories followed a similar trend (Supplementary Figure S6B and C). In these conditions we obtained a slightly lower *K*_D_ value (1.1 ± 0.1 mM) and a similar Hill coefficient (2.2 ± 0.4) (Supplementary Figure S6A). These data confirm that K^+^ only plays a role during the metabolite-recognition phase by mediating lysine–RNA interactions, as observed in the crystal structure of the ligand-bound aptamer.

smFRET analysis of two aptamer variants, L2X and L4X, which disrupt the formation of the L2–L3 and P2–L4 interactions (18), respectively (Supplementary Figure S7A and Supplementary Table S1), confirmed that the stabilization of the ligand-free folded state (**F_LF_**) requires the formation of both tertiary contacts (Supplementary Figure S7b). Interestingly, an aptamer variant carrying a G39C substitution that was expected to only abolish ligand binding (BPX) (21) also severely compromised the formation of the **F_LF_** state (Supplementary Figure S7B), suggesting that the binding pocket becomes at least partially structured in that state.

### Metabolite sensing by unstructured *lysC* aptamers

Next, we investigated whether the ligand could directly interact with aptamers in the **U** state. In a background of 0.5 mM Mg^2+^, where the aptamer is exclusively in the **U** state (Figure 2A, upper panel), we observed a progressive increase in the contribution of a new high-FRET state as we increased the ligand concentration. In 300 μM lysine, the relative contribution of this high-FRET state is ∼42% (Figure 2C, upper panel and Supplementary Figure S8A). When we repeated these experiments in 1 mM Mg^2+^, we observed an increase in ligand binding affinity. We found that the addition of only 75 μM lysine ligand was enough to induce a shift of ∼65% of the *lysC* aptamer population into the high-FRET state (Figure 2D, bottom panel and Supplementary Figure S9a). This high-FRET population was centred at *E*_app_ = 0.66 ± 0.01, in good agreement with the modelled distance (Figure 1C) and higher than the FRET efficiency of the **F_LF_** state (*E*_app_ ∼ 0.6) (Figure 2E), so we designated it the ligand-bound folded state, **F_LB_**.

smFRET trajectories obtained in 0.5 mM Mg^2+^ and increasing lysine concentrations exhibited only fluctuations between **U** and **F_LF_** (Figure 2D, upper panel and Supplementary Figure S8B). In 1 mM Mg^2+^, the smFRET traces showed shorter dwell times in **U**, reflecting the shift in the equilibrium populations towards the **F_LB_** state (Figure 2D, bottom panel and Supplementary Figure S9). We concluded that ligand binding to tertiary-unstructured aptamers induces the direct transition to the ligand-bound folded state, **F_LB_**, distinguished from **F_LF_** by at least a closer juxtaposition of stems P1 and P5. The fractional occupancies of the **U** and **F_LB_** states as a function of the lysine concentration were extracted by calculating the normalized areas of the **U** and **F_LB_** Gaussian populations at each ligand concentration (Figure 2F). Fitting the fractional occupancies to a two-state binding model yielded *K*_D_ values of 180 ± 20 μM and 33 ± 3 μM at 0.5 mM and 1 mM Mg^2+^, respectively.

### Ligand-induced dynamics in unstructured *lysC* aptamers

**Figure 3.**
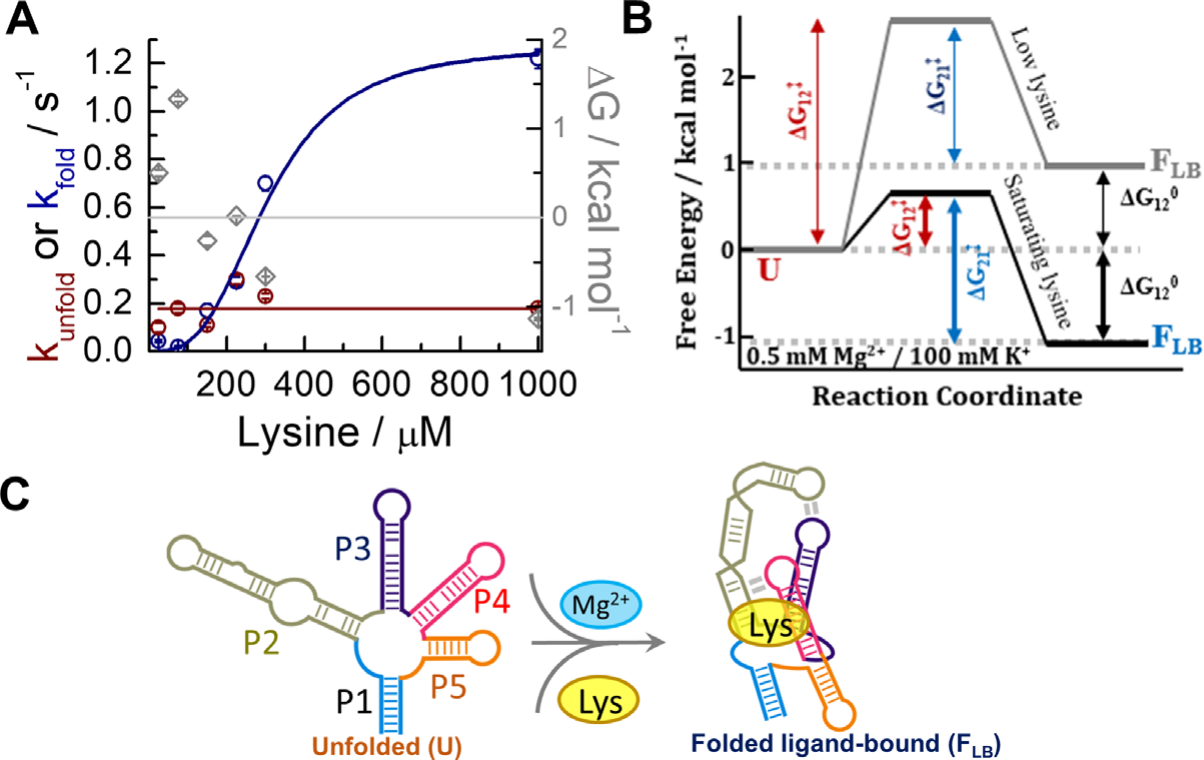
Ligand binding to unstructured aptamers promotes direct folding into the native state. (**A**) Kinetic rates obtained for the **U**→ **F_LB_** (dark blue) and **F_LB_** →**U** (dark red) transitions as a function of lysine concentration in a background of 100 mM K^+^ and 0.5 mM Mg^2+^. The solid lines represent fits to a straight line for the **F_LB_** →**U** transition, and to a Hill model for the **U**→ **F_LB_** transition. Free energy values in kcal/mol obtained from the kinetics rates are also plotted (◊, grey), and Δ*G*° = 0 is indicated by a solid grey line. (**B**) Free energy landscape of the *lysC* aptamer in 0.5 mM Mg^2+^ ions at low (∼25 μM, grey line) and saturating lysine concentrations (1 mM, black line). The diagram shows the free energy differences (Δ*G*°) and barrier heights (Δ*G*^‡^) between states **U** (denoted state 1 in subscripts for simplicity) and **F_LB_** (denoted state 2). The absolute barrier heights are representative only but have been scaled such that the magnitudes of the relevant changes ΔΔ*G*^‡^ (Supplementary Table S6) are accurately represented. (**C**) Single-step mechanism proposed for the interplay between folding and induced-fit ligand binding in the *lysC* aptamer at sub-saturating concentrations of Mg^2+^ (<1 mM).

We quantified the dynamics of **U**↔**F_LB_** transitions by calculating the distribution of dwell times in the **U** and **F_LB_** states as a function of lysine concentration (Figure 3A, Supplementary Figure S10 and Supplementary Table S2). In 0.5 mM Mg^2+^, the rate of the **F_LB_→U** transition was independent of lysine concentration, and when fitted to a straight line, it yielded a value of 0.18 ± 0.03 s^−1^ (Supplementary Table S3). In contrast, the **U→F_LB_** docking rate increased by ∼60-fold, from 0.019 ± 0.003 s^−1^ in 75 μM lysine to 1.22 ± 0.04 s^−1^ in 1 mM lysine (Supplementary Table S2). The association rates were fitted to a two-state model (Supplementary Equation 2) and yielded a dissociation constant of 310 ± 50 μM and a Hill coefficient of 3.1 ± 0.7, implying a strong coupling between ligand binding and aptamer folding at sub-saturating Mg^2+^ concentrations.

To investigate this mechanism in more detail, we calculated the free energy differences between structural states (Δ*G*°) and the changes in the barrier height (ΔΔ*G*^‡^) from the single-molecule kinetic data (Figure 3B). In 0.5 μM lysine, **U** is more energetically favourable than **F_LB_ (**Δ*G*° = 0.89 ± 0.02 kcal/mol) (Supplementary Table S3). This scenario reverses at high ligand concentrations. For instance, in 150 μM lysine, **F_LB_** is 1.1 ± 0.1 kcal/mol more favourable than **U**, and the height of the energy barrier for docking and folding has decreased by ∼1.9 ± 0.1 kcal/mol, while the dissociation energy barrier has changed only by –0.34 ± 0.08 kcal/mol. A ligand-dependent docking rate coupled with a ligand-independent unfolding rate fulfills the canonical definition of an induced-fit ligand-binding mechanism (Figure 3C), in which lysine binding re-structures the junction, promotes L2–L3 and P2–L4 contacts, and reorients the P1 and P5 stems, thus orchestrating the formation of the global tertiary structure.

### Metabolite sensing by partially-folded *lysC* aptamers

**Figure 4.**
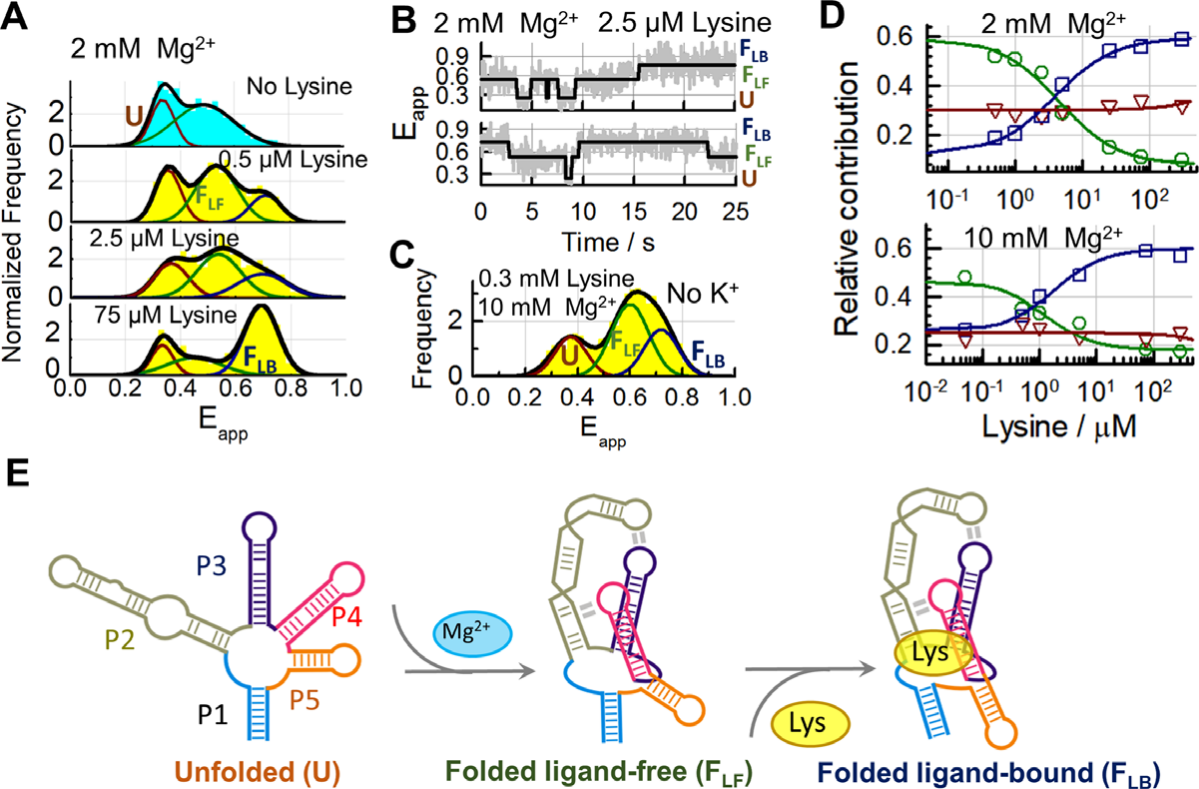
Ligand-induced re-organization of tertiary pre-organized lysine aptamers (**A**) Single-molecule FRET histograms obtained in the absence of ligand (cyan) and at the indicated concentrations of lysine ligand (yellow) in a background of 100 mM K^+^ and 2 mM Mg^2+^. The result from fitting each histogram to three Gaussians (black line) and the relative contributions of the **U** (red), **F_LF_** (green) and **F_LB_** (blue) states are also shown. (**B**) Representative FRET trajectories obtained in 2 mM Mg^2+^, 100 mM K^+^ and 2.5 μM lysine showing fluctuations between **U, F_LF_** and **F_LB_** states within single aptamers. The solid line represents the idealized FRET trajectory obtained using Hidden Markov modelling. (**C**) Single-molecule FRET histogram obtained in the absence of K^+^ ions at 10 mM Mg^2+^ ions and 300 μM lysine. (**D**) Variation in the relative contributions of **U** (Δ, red), **F_LF_** (o, green), and **F_LB_** (□, blue) states as a function of lysine concentration obtained in a background of 100 mM K^+^ at 2 mM (upper panel) and 10 mM (bottom panel) Mg^2+^. The solid lines represent the result of globally fitting the contributions of the **F_LF_** and **F_LB_** states as a function of lysine ligand concentration to a Hill model (Supplementary Methods). Dissociation constants of 4.5 ± 1 μM and 1.3 ± 0.5 μM were obtained in 2 mM and 10 mM Mg^2+^, respectively. (**E**) Proposed two-step folding and ligand-binding mechanism for the lysC aptamer at saturating concentrations of Mg^2+^.

Our analysis of the folding of the *lysC* aptamer revealed that the **F_LF_** state can be efficiently populated at the high end of the physiological Mg^2+^ concentration range (∼2 mM). We hypothesised that **F_LF_** might function as a high-affinity binding scaffold for lysine in these conditions. To test this hypothesis, we titrated the aptamer with lysine in a background of 2 mM (Figure 4A) or 10 mM (Supplementary Figure S11) Mg^2+^. The smFRET histograms showed that as the lysine concentration increased, a third Gaussian population appeared centred at *E*_app_ ∼ 0.69 ± 0.01, consistent with the **F_LB_** state. With a difference in *E*_app_ of 0.09 ± 0.01, the **F_LB_** and **F_LF_** states can be clearly distinguished in single-molecule traces (Figure 3B). The relative contribution of the **F_LB_** population in 75 μM lysine and 2 mM Mg^2+^was 59% (Figure 4A). Increasing the Mg^2+^ concentration to 10 mM only increased the fractional occupancy to 64% (Supplementary Figure S11), indicating that the **F_LB_** state is already efficiently formed in physiological Mg^2+^ concentrations.

Analysis of the tertiary structure variants, L2X and L4X, and the binding knockout aptamer, BPX, confirmed that none of the aptamer variants can adopt the **F_LB_** conformation, even in high concentrations of Mg^2+^ or lysine (Supplementary Figure S12). Furthermore, the native aptamer cannot form the **F_LB_** state efficiently when K^+^ is omitted or substituted with Na^+^ (Supplementary Figure S13), even in lysine concentrations as high as 300 μM (Figure 4c). This confirms the specific role of K^+^ ions in stabilizing key RNA–ligand interactions.

We plotted the fractional occupancies of each state (**U, F_LF_** and **F_LB_**) as a function of lysine in both 2 mM and 10 mM Mg^2+^ (Figure 4D). The fractional occupancy of the **U** state was higher in 2 mM (∼35%) than in 10 mM Mg^2+^ (∼25%), but it remained nearly constant when increasing the concentration of lysine, while the **F_LF_** and **F_LB_** states exchanged populations. A global fitting of the binding isotherms for these two states yielded *K*_D_ values of 3 ± 1 μM in 2 mM and 1.3 ± 0.5 μM in 10 mM Mg^2+^, two orders of magnitude lower than in 0.5 mM Mg^2+^ (∼310 ± 50 μM). Omitting K^+^ ions from the medium increased the *K*_D_ to 850 ± 35 μM (Supplementary Figure S14), comparable to the value of 660 ± 50 μM reported by Batey *et al.* using a 2-aminopurine fluorescence assay (22). As summarized in Figure 4E, at sufficiently high concentrations of Mg^2+^, the aptamer domain is mostly organized into the **F_LF_** state, in which the L2–L3 and P2–L4 contacts are formed. Lysine binding to **F_LF_** ‘zips up’ the binding pocket by coordinating the two base pairs at the top of the P1 stem and promoting the stacking of P1/P2 and P4/P5 as observed in the crystal structure.

### Conformational dynamics of partially-folded *lysC* aptamers

Our analysis of the single-molecule equilibrium populations in 2 mM Mg^2+^ revealed the coexistence of the **U, F_LF_** and **F_LB_** states in the presence of lysine ligand and physiologically relevant Mg^2+^ concentrations (Figure 4A). To characterize the interconversion kinetics between these states, we examined the dynamics of single aptamers in 2 mM Mg^2+^ ions and 2.5 μM lysine ligand. In these conditions, all three states are populated (Figure 4A), and transitions between them are regularly observed in smFRET trajectories (Figure 4B).

**Figure 5.**
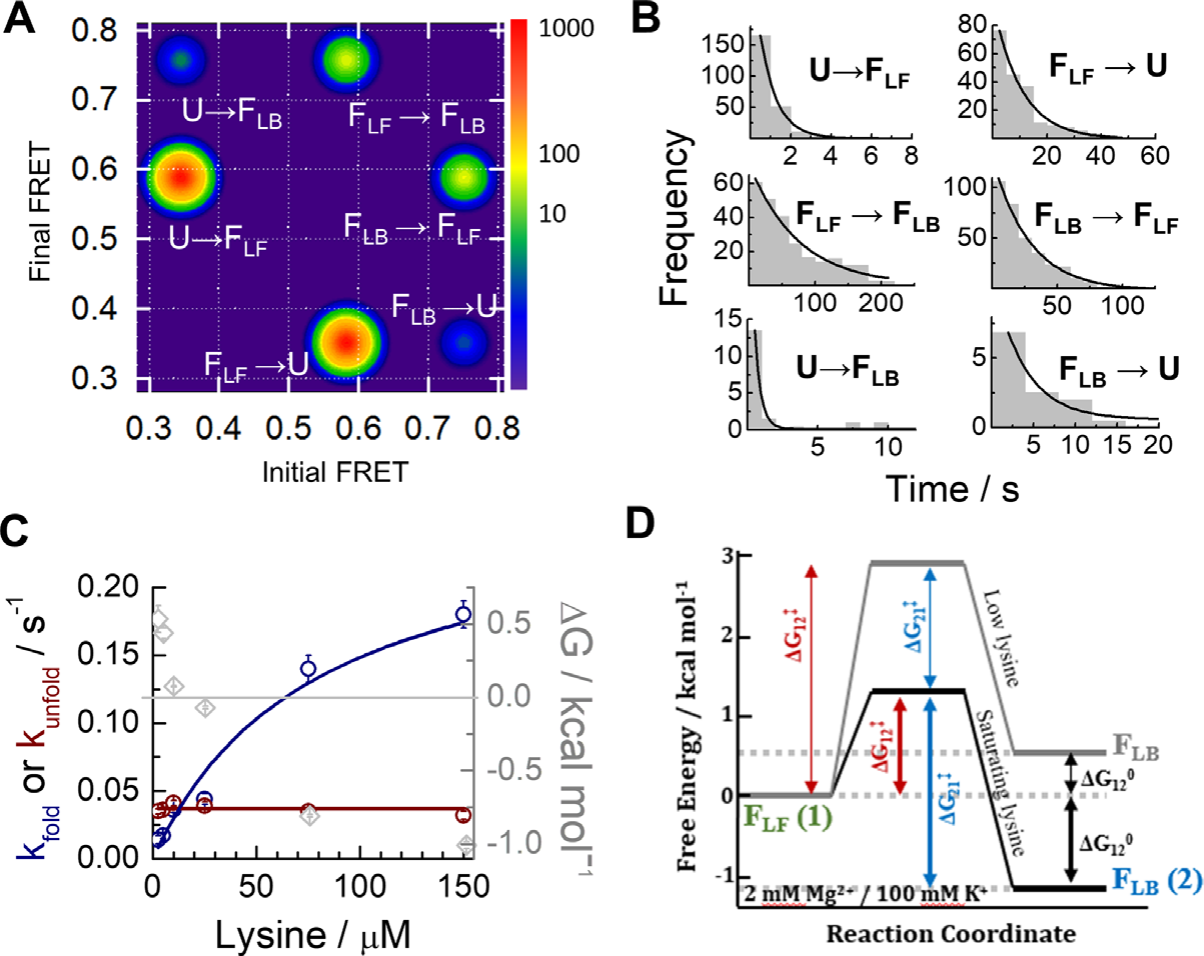
Single-molecule kinetic analysis confirms an induced-fit model for ligand binding at all experimental conditions. (**A**) Transition density plot (TDP) obtained in 2 mM Mg^2+^, 100 mM K^+^ and 2.5 μM lysine, showing the number of times a given transition from *E*_initial_ to *E*_final_ was identified in FRET traces showing dynamics. The TDP indicates a prevalence of **U** → **F_LF_** and **F_LF_** → **F_LB_** transitions over direct **U** ↔**F_LB_** dynamics. (**B**) Single-molecule dwell-time histograms for each type of FRET transition shown in the TDP. The solid line represents the fit to a mono-exponential decay function. (**C**) Kinetic rates for the interconversion between the **F_LF_** and **F_LB_** states obtained as a function of lysine concentration. The **F_LB_** → **F_LF_** rate (dark red) stays constant at 0.037 ± 0.001 s^−1^, while the **F_LF_** → **F_LB_** rate (blue) increases with ligand concentration. The free energy difference between **F_LF_** and **F_LB_**, Δ*G*° (grey), is also plotted. The **F_LB_** state becomes energetically favourable (Δ*G*° < 0) around 10 μM lysine concentration. (**D**) Free energy differences Δ*G*° and barrier heights Δ*G*^‡^ between **F_LF_** (denoted state 1 for clarity) and **F_LB_** (state 2). The grey line represents the landscape at 2 mM Mg^2+^ and 2.5 μM lysine and the black line represents the landscape in 300 μM lysine. The absolute barrier heights are representative only but have been scaled such that the magnitudes of the relevant changes ΔΔ*G*^‡^ (Supplementary Table S6) are accurately represented.

A transition-density plot (TDP) of the raw number of FRET transitions revealed six populations corresponding to pairwise transitions between the **U, F_LF_** and **F_LB_** states (Figure 5a and Supplementary Table S4. Note the logarithmic intensity scale in Figure 5A). The statistical predominance of the folding route involving all three states (**U**↔**F_LF_**↔**F_LB_**) over direct **U**↔ **F_LB_** transitions can be clearly observed. In the ∼450 transitions analysed in which **U** was the initial state, less than 10% of them involved a direct **U**↔**F_LB_** transition (Supplementary Table S4). We extracted the six kinetic rates involved in the **U**↔**F_LF_**↔**F_LB_** mechanism in 2.5 μM lysine by fitting the dwell-time histogram of each transition with a mono-exponential decay function (Figure 5b and Supplementary Table S4). The undocking transition **F_LB_→ F_LF_** was 7-fold slower (0.036 ± 0.003 s^−1^) than the rare **F_LB_** → **U** transition (0.25 ± 0.06 s^−1^). In contrast, direct docking via the **U**→ **F_LB_** route, although infrequent, was 150-fold faster (2.3 ± 0.3 s^−1^) than docking through the **F_LF_**→**F**_**LB**_ pathway (0.014 ± 0.003 s^−1^) (Figure 5B and Supplementary Table S4). This suggests that complexes form faster via the direct two-state pathway (**U**↔**F_LB_**), but this route leads to a much less stable **F_LB_** state than when formed through the **F_LF_** intermediate.

When we extended the kinetic analysis over a lysine concentration range spanning more than two orders of magnitude (Figure 5C and Supplementary Table S5), we found that the unfolding rate was independent of ligand concentration, whereas the **F_LF_**→**F_LB_** docking rate increased by a factor of 10 and became similar to that observed for the **U**→**F**_LB_ transition (Figure 5B). Fitting this ligand-dependent rate (Supplementary Eq. 2) yielded a *K*_D_ of 80 ± 30 μM and an intrinsic *k*_fold_ for the **F_LF_**→**F**_**LB**_ transition of 0.27 ± 0.08 s^−1^. Using this *k*_fold_ and the **F_LB_→ F_LF_** rate obtained previously, we determined the equilibrium constant for the ligand-induced conformational change and obtained a value of 0.14 ± 0.04. The apparent *K*_D_ of the entire transition (*K*_D, app_) was determined from the product *K*_D, fold_• *K*_D, bind_ to be 11 ± 7 μM, close to the value of 3 ± 1 μM obtained by fitting the fractional occupancies of **F_LF_** and **F_LB_** (Figure 4D, upper panel).

The free energy differences between the **F_LF_** and **F_LB_** states calculated from the kinetic rates (Figure 5B–D and Supplementary Table S6) suggest that the ligand ‘tilts’ the energy landscape of the aptamer–ligand encounter complex such that **F_LB_** becomes more favourable by –1.84 ± 0.05 kcal/mol. The energy barrier for transitions from **F_LF_** to **F_LB_** also decreases by 1.49 ± 0.09 kcal/mol, whilst the barrier to complex dissociation has increased relative to the low-Mg^2+^ case due to Mg^2+^ stabilization of the complex. However, it remained insensitive to lysine concentration (ΔΔ*G*^‡^ = +0.05 ± 0.01 kcal/mol). Thus, although Mg^2+^ ions pre-organize much of the tertiary structure of the aptamer, the metabolite-binding **F_LF_**→**F**_**LB**_ transition retains the kinetic features of an induced-fit mechanism.

## DISCUSSION

In this study, we have revealed that the nature of the RNA-ligand encounter complex, the ligand-binding affinity, the folding pathway and the regulatory function of the *lysC* riboswitch (Figure 1) are all strongly modulated by small variations within the physiological range (∼0.5–3 mM) of Mg^2+^ concentrations (Figures 2–4). Mg^2+^-induced compaction of the RNA structure or organization of a native or near-native state are common themes in riboswitch folding (39,42). However, the observed sensitivity of the *lysC* aptamer's structure and ligand-binding affinity to small changes within the physiological Mg^2+^ concentration range is more nuanced than simple Mg^2+^-induced compaction. Our results indicate that Mg^2+^ actively tunes the structure of the ligand-free state to produce encounter complexes, **U** and **F_LF_**, with strikingly different sensitivities to the ligand (Figures 2F and 4D) in different, yet equally biologically accessible (Supplementary Figure S1), environments (37).

Our single-molecule analysis also demonstrates that the formation of the **F_LF_** state and the associated increase in binding affinity to the low micromolar regime are not artefacts of saturating Mg^2+^ concentrations, as concluded in a previous study (34). Rather, the observed two-order-of-magnitude change in binding affinity reflects a Mg^2+^-induced shift between these two distinct and biologically relevant binding-competent structures, **U** and **F_LF_**.

Absolute metabolite concentrations have been reported in *E. coli* using liquid-chromatography-tandem mass spectrometry (43). The intracellular concentration of lysine found in glucose-fed exponentially growing *E. coli* was 400 μM, which is within the lysine concentration range employed in our studies, and orders of magnitude lower than the concentration at which the amino acid lysine has been recently shown to play a non-specific role in RNA stabilization (35,44). Thus, our single-molecule data collected in the 0.5–2 mM Mg^2+^ range with lysine concentrations up to 1 mM constitute a reasonable model of the extrema of *in vivo* function in *B. subtilis*, and the observed tunability of *lysC* function by Mg^2+^ and lysine ligand represents an intrinsic feature of its regulatory mechanism. On the other hand, the intracellular K^+^ concentration in *B. subtilis* is in the range 200–400 mM (45,46), and K^+^ ions have been shown to coordinate lysine aptamer folding at concentrations as low as 10 mM (22). Accordingly, in the following discussion, we treat the K^+^ background as effectively saturating, which it would be in any but the most extreme environmental conditions.

The observed kinetics of ligand binding suggest that the *lysC* riboswitch regulates gene expression on a Mg^2+^-tunable scale between kinetic and thermodynamic control. For instance, in 2 mM Mg^2+^ and 300 μM lysine, its dwell times in the **F_LB_** and **F_LF_** states are 27 ± 2 s and 2.8 ± 0.3 s, respectively (Supplementary Table S6). The time required to transcribe the expression platform without pausing is 1–3 s at bacterial transcription speeds of 20–60 nt/s (47). An unfolded-state dwell time on the order of the transcription speed is the hallmark of kinetic control, but a folded-state dwell time longer than the transcription time is characteristic of thermodynamic control. If the aptamer does bind the ligand within the transcription window, it is unlikely to unfold while the transcription machinery is still bound. However, as the Mg^2+^ concentration decreases, the unfolding rate increases (Supplementary Table S3), becoming comparable to the transcription time and shifting the gene regulatory dynamics of the aptamer closer to kinetic control. Based on our results, we expect that the riboswitch will be able to bind physiological concentrations of lysine in any environment, but that increasing the Mg^2+^ concentration increases the fidelity of the gene-regulatory signal by accentuating the thermodynamic-like features of the regulatory mechanism.

Ligand-binding mechanisms are broadly classified as following either a ‘conformational selection’ or an ‘induced fit’ folding pathway (48,49). These pathways can be defined either structurally, in terms of whether the ligand binds after or before folding of the *holo* state; or kinetically, in terms of whether ligand binding reduces the unfolding rate of the *holo* state (selection) or increases the folding rate (induction). Many of the riboswitches studied using single-molecule microscopy to date have been shown to pre-organize into a native or native-like state in the presence of Mg^2+^ but the absence of ligand (47,49–58).

This intrinsic Mg^2+^-induced compaction has led to the suggestion that ligand binding to riboswitches in general stabilizes (‘selects’) the native state from a pre-existing manifold. However, it is logically possible that a riboswitch could exhibit traits of either mechanism, according to either definition, and that these ‘mixed’ mechanisms could be environmentally dependent. For example, the Pre-Q_1_ aptamer transitions from an induced fit mechanism to conformational selection as a result of cation-induced folding (56), while the *env8* hydroxocobalamin (HyCbl) riboswitch shows binding of the ligand to an unfolded state of the aptamer as expected of induced fit, but also the characteristic slowing of the unfolding rate with increasing HyCbl concentration that indicates conformational selection (59). The manganese-sensing riboswitch *yybP-ykoY* from *B. subtilis* has also been shown to shift between induced-fit and conformational selection pathways in response to cellular levels of Mg^2+^ (60).

**Figure 6.**
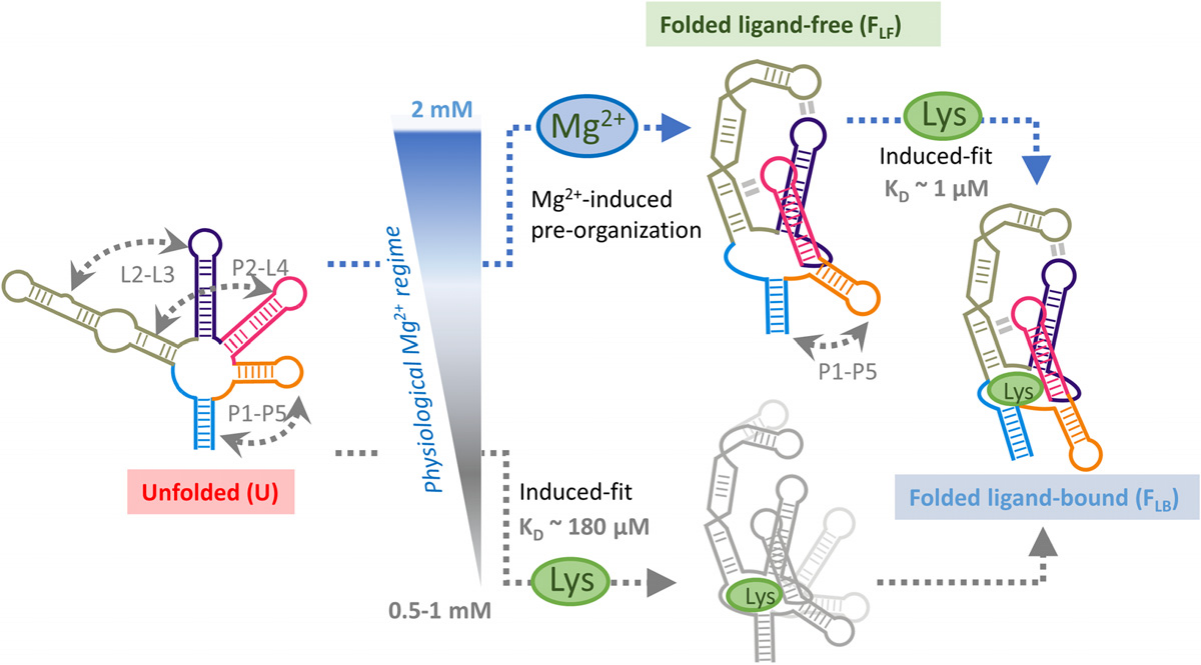
The folding and ligand-binding mechanism of the lysine aptamer is controlled by a narrow change in the physiological concentration of Mg^2+^ ions. Schematic of the interplay between folding and induced-fit ligand binding in the *lysC* aptamer, emphasising the structural changes taking place within a very narrow Mg^2+^ concentration window and the alternative folding routes predominant at each condition. At the low end of the physiological Mg^2+^ concentration range (<1 mM), the aptamer remains unstructured, and the encounter complex needs to form most tertiary contacts with the assistance of the ligand (lower route). An increase of just 1 mM in the concentration of Mg^2+^ ions is enough to shift the aptamer to a tertiary pre-organised structure (upper route). The encounter complex evolves into the native state by shortening the P1–P5 distance and closing the ligand-binding pocket. The lysine aptamer uses a dual-input signalling model to switch between a single-step, poor-affinity pathway at low concentrations of Mg^2+^ ions to a high-affinity, two-step mechanism at high physiological concentrations.

Growing evidence suggests that ‘mixed’ ligand-recognition mechanisms with features of both conformational selection and induced fit are common, perhaps even prevalent, in ligand binding to RNA (49). However, our single-molecule population distribution analysis (Figure 3a-b) and kinetic data (Figure 5c-d) support a model where the *lysC* aptamer recognizes the ligand exclusively using an induced-fit mechanism (Figure 6) in both the structural and kinetic senses. To our knowledge, it is the first example of a natural aptamer that does so across the whole physiological range of Mg^2+^ concentrations. This fact is all the more remarkable given that the Mg^2+^-induced compaction widespread among riboswitches commonly organises the *holo* state directly, as in the case of Pre-Q_1_ (56), the *add* and *pbuE* adenine riboswitches (52,61–62), and many others (63,64). In the case of *lysC*, Mg^2+^ tunes the affinity of the aptamer for lysine but switching still occurs exclusively in the presence of the ligand.

Other examples of environmental dependence exist among riboswitches (51,61–62). For example, the *add* riboswitch from *V. vulnificus* uses temperature-dependent switching between two ligand-free secondary structures to modulate its response to adenine inside and outside its host (61,62). The SAM-I riboswitch from *T. tengcongensis* achieves maximal aptamer preorganisation in the physiological Mg^2+^ concentration range, while higher concentrations favour the organisation of the expression platform (64). The *lysC* aptamer's dynamic dependence on Mg^2+^ concentration variations within the physiological range can be considered a new type of environmental modulation, in which Mg^2+^ tunes the ligand affinity of the aptamer and the fidelity of its regulatory outcome by varying the structure of the encounter complex without inducing *holo* state formation (51). *In vivo* experiments linking the expression of *lysC* to the variation in the concentrations of Mg^2+^ and ligand in both the normal cell cycle and stressed environments is needed to illuminate the biological role of this novel dual-layer control mechanism.

In summary, we have demonstrated the first example of an RNA aptamer that has optimized its response to Mg^2+^ ions to act as a fine-tuning element of its tertiary structure. By doing so, it modulates its affinity for its cognate ligand by two orders of magnitude without the need to alter its nucleotide sequence, a strategy that might also be exploited by other regulatory elements to tightly control gene expression.

## Supplementary Material

gkz316_Supplemental_File

## Notes

Present address: Kaley McCluskey, Department of Bionanoscience, Delft University of Technology, 2629 HZ.Delft, The Netherlands.

Present address: Cibran Perez-Gonzalez, Centro Singular the Investigation en Quimica Biologica y Materiales Moleculares, Universidad de Santiago de Compostela, Santiago de Compostela 15782, Spain.

## References

[1] Irnov A.K., Winkler W.C. Genetic control by cis- acting regulatory RNAs in Bacillus subtilis: general principles and prospects for discovery. Cold Spring Harb. Symp. Quant. Biol. 2006; 71:239–249. 17381303 10.1101/sqb.2006.71.021

[2] Montange R.K., Batey R.T. Riboswitches: emerging themes in RNA structure and function. Annu. Rev. Biophys. 2008; 37:117–133. 18573075 10.1146/annurev.biophys.37.032807.130000

[3] Sherwood A. V., Henkin T.M. Riboswitch-mediated gene regulation: novel RNA architectures dictate gene expression responses. Annu. Rev. Microbiol. 2016; 70:361–374. 27607554 10.1146/annurev-micro-091014-104306

[4] Mellin J.R., Cossart P. Unexpected versatility in bacterial riboswitches. Trends Genet. 2015; 31:150–156. 25708284 10.1016/j.tig.2015.01.005

[5] Mandal M., Boese B., Barrick J.E., Winkler W.C., Breaker R.R. Riboswitches control fundamental biochemical pathways in *Bacillus subtilis* and other bacteria. Cell. 2003; 113:577–586. 12787499 10.1016/s0092-8674(03)00391-x

[6] Jones C.P., Ferré-D’Amaré A.R. Long-Range interactions in riboswitch control of gene expression. Annu. Rev. Biophys. 2017; 46:455–481. 28375729 10.1146/annurev-biophys-070816-034042 PMC7374767

[7] Cheah M.T., Wachter A., Sudarsan N., Breaker R.R. Control of alternative RNA splicing and gene expression by eukaryotic riboswitches. Nature. 2007; 447:497–501. 17468745 10.1038/nature05769

[8] Serganov A., Patel D.J. Amino acid recognition and gene regulation by riboswitches. BBA - Gene Regul. Mech. 2009; 1789:592–611. 10.1016/j.bbagrm.2009.07.002 PMC3744886 19619684

[9] Sudarsan N., Wickiser J.K., Nakamura S., Sudarsan N., Wickiser J.K., Nakamura S., Ebert M.S., Breaker R.R. An mRNA structure in bacteria that controls gene expression by binding lysine. Genes Dev. 2003; 17:2688–2697. 14597663 10.1101/gad.1140003 PMC280618

[10] Mandal M., Lee M., Barrick J.E., Weinberg Z., Emilsson G.M., Ruzzo W.L., Breaker R.R. A glycine-dependent riboswitch that uses cooperative binding to control gene expression. Science. 2004; 306:275–279. 15472076 10.1126/science.1100829

[11] Ren A., Xue Y., Peselis A., Serganov A., Al-Hashimi H.M., Patel D.J. Structural and dynamic basis for low-affinity, high-selectivity binding of L-glutamine by the glutamine riboswitch. Cell Rep. 2015; 13:1800–1813. 26655897 10.1016/j.celrep.2015.10.062 PMC4690532

[12] Wilson-Mitchell S.N., Grundy F.J., Henkin T.M. Analysis of lysine recognition and specificity of the Bacillus subtilis L box riboswitch. Nucleic Acids Res. 2012; 40:5706–5717. 22416067 10.1093/nar/gks212 PMC3384330

[13] Mukherjee S., Barash D., Sengupta S. Comparative genomics and phylogenomic analyses of lysine riboswitch distributions in bacteria. PLoS One. 2017; 12:1–24. 10.1371/journal.pone.0184314 PMC5584792 28873470

[14] Caron M.-P., Bastet L., Lussier A., Simoneau-Roy M., Masse E., Lafontaine D.A. Dual-acting riboswitch control of translation initiation and mRNA decay. Proc. Natl. Acad. Sci. U.S.A. 2012; 109:E3444–E3453. 23169642 10.1073/pnas.1214024109 PMC3528543

[15] Blount K.F., Wang J.X., Lim J., Sudarsan N., Breaker R.R. Antibacterial lysine analogs that target lysine riboswitches. Nat. Chem. Biol. 2006; 3:44. 17143270 10.1038/nchembio842

[16] Hallberg Z.F., Su Y., Kitto R.Z., Hammond M.C. Engineering and in vivo applications of riboswitches. Annu. Rev. Biochem. 2017; 86:515–539. 28375743 10.1146/annurev-biochem-060815-014628

[17] Blouin S., Chinnappan R., Lafontaine D.A. Folding of the lysine riboswitch: importance of peripheral elements for transcriptional regulation. Nucleic Acids Res. 2011; 39:3373–3387. 21169337 10.1093/nar/gkq1247 PMC3082890

[18] Blouin S., Lafontaine D.A. A loop loop interaction and a K-turn motif located in the lysine aptamer domain are important for the riboswitch gene regulation control. RNA. 2007; 13:1256–1267. 17585050 10.1261/rna.560307 PMC1924893

[19] Garst A.D., Heroux A., Rambo R.P., Batey R.T. Crystal structure of the lysine riboswitch regulatory mRNA. J. Biol. Chem. 2008; 283:22347–22351. 18593706 10.1074/jbc.C800120200 PMC2504901

[20] Serganov A., Huang L., Patel D.J. Structural insights into amino acid binding and gene control by a lysine riboswitch. Nature. 2008; 455:1263–1268. 18784651 10.1038/nature07326 PMC3726722

[21] Smith-Peter E., Lamontagne A.-M., Lafontaine D.A. Role of lysine binding residues in the global folding of the lysC riboswitch. RNA Biol. 2015; 12:1372–1382. 26403229 10.1080/15476286.2015.1094603 PMC4829333

[22] Garst A.D., Porter E.B., Batey R.T. Insights into the regulatory landscape of the lysine riboswitch. J. Mol. Biol. 2012; 423:17–33. 22771573 10.1016/j.jmb.2012.06.038 PMC3444622

[23] Baird N.J., Ferre-D’Amare A.R. Idiosyncratically tuned switching behavior of riboswitch aptamer domains revealed by comparative small-angle X-ray scattering analysis. RNA. 2010; 16:598–609. 20106958 10.1261/rna.1852310 PMC2822924

[24] McCluskey K., Shaw E., Lafontaine D.A., Penedo J.C. Engelborghs Y, Visser JWG Single-molecule fluorescence of nucleic acids. Methods in Molecular Biology. 2013; 1076:Totowa Humana Press 759–791. 10.1007/978-1-62703-649-8_35 24108654

[25] Heppell B., Mulhbacher J., Penedo J.C., Lafontaine D.A. Application of fluorescent measurements for the characterization of riboswitch-ligand interactions. Methods Mol. Biol. 2009; 540:25–37. 19381550 10.1007/978-1-59745-558-9_3

[26] Roy R., Hohng S., Ha T. A practical guide to single-molecule FRET. Nat.Methods. 2008; 5:507–516. 18511918 10.1038/nmeth.1208 PMC3769523

[27] Spizizen J. Transformation of biochemically deficient strains of Bacillus subtilis by deoxyribonucleate. Proc. Nat. Acad. Sci. U.S.A. 1958; 44:1072–1078. 10.1073/pnas.44.10.1072 PMC528696 16590310

[28] Darnajoux R., Constantin J., Miadlikowska J., Lutzoni F., Bellenger J.P. Is vanadium a biometal for boreal cyanolichens. New Phytol. 2014; 202:765–771. 24641550 10.1111/nph.12777

[29] Boudreault J.D., Perez-Gonzalez C., Penedo J.C., Lafontaine D.A. Single-molecule approaches for the characterization of riboswitch folding mechanisms. Methods Mol. Biol. 2015; 1334:1–347. 26404145 10.1007/978-1-4939-2877-4_6

[30] Rasnik I., McKinney S.A., Ha T. Nonblinking and long-lasting single-molecule fluorescence imaging. Nat. Methods. 2006; 3:891–893. 17013382 10.1038/nmeth934

[31] Aitken C.E., Marshall R.A., Puglisi J.D. An oxygen scavenging system for improvement of dye stability in single-molecule fluorescence experiments. Biophys. J. 2008; 94:1826–1835. 17921203 10.1529/biophysj.107.117689 PMC2242739

[32] Perez-Gonzalez C., Lafontaine D.A., Penedo J.C. Fluorescence-Based strategies to investigate the structure and dynamics of aptamer-ligand complexes. Front. Chem. 2016; 4:1–22. 27536656 10.3389/fchem.2016.00033 PMC4971091

[33] Garst A.D., Héroux A., Rambo R.P., Batey R.T. Crystal structure of the lysine riboswitch regulatory mRNA element. J. Biol. Chem. 2008; 283:22347–22351. 18593706 10.1074/jbc.C800120200 PMC2504901

[34] Fiegland L.R., Garst A.D., Batey R.T., Nesbitt D.J. Single-molecule studies of the lysine riboswitch reveal effector-dependent conformational dynamics of the aptamer domain. Biochemistry. 2012; 51:9223–9233. 23067368 10.1021/bi3007753 PMC3703957

[35] Yamagami R., Bingaman J.L., Frankel E.A., Bevilacqua P.C. Cellular conditions of weakly chelated magnesium ions strongly promote RNA stability and catalysis. Nat. Commun. 2018; 9:1–12. 29858572 10.1038/s41467-018-04415-1 PMC5984629

[36] Froschauer E.M., Kolisek M., Dieterich F., Schweigel M., Schweyen R.J. Fluorescence measurements of free [Mg2+] by use of mag-fura 2 in Salmonella enterica. FEMS Microbiol. Lett. 2004; 237:49–55. 15268937 10.1016/j.femsle.2004.06.013

[37] Lemay K.A., Assmann S.M., Mathews D.H., Bevilacqua P.C. Bridging the gap between in vitro and in vivo RNA folding. Q. Rev. Biophys. 2016; 49:e10. 27658939 10.1017/S003358351600007X PMC5269127

[38] St-Pierre P., McCluskey K., Shaw E., Penedo J.C., Lafontaine D.A. Fluorescence tools to investigate riboswitch structural dynamics. Biochim. Biophys. Acta - Gene Regul. Mech. 2014; 1839:1005–1019. 10.1016/j.bbagrm.2014.05.015 24863161

[39] Borner R., Kowerko D., Miserachs H.G., Schaffer M.F., Sigel R.K.O. Metal ion induced heterogeneity in RNA folding studied by smFRET. Coord. Chem. Rev. 2016; 327–328:123–142.

[40] Morten M.J., Peregrina J.R., Figueira-Gonzalez M., Ackermann K., Bode B.E., White M.F., Penedo J.C. Binding dynamics of a monomeric SSB protein to DNA: A single-molecule multi-process approach. Nucleic Acids Res. 2015; 43:10907–10924. 26578575 10.1093/nar/gkv1225 PMC4678828

[41] Kalinin S., Peulen T., Sindbert S., Rothwell P.J., Berger S., Restle T., Goody R.S., Gohlke H., Seidel C.A.M. A toolkit and benchmark study for FRET-restrained high-precision structural modeling. Nat. Methods. 2012; 9:1218–1227. 23142871 10.1038/nmeth.2222

[42] Lemay J.-F., Penedo J.C., Mulhbacher J., Lafontaine D.A. Molecular basis of RNA-mediated gene regulation on the adenine riboswitch by single-molecule approaches. Methods Mol. Biol. 2009; 540:65–76. 19381553 10.1007/978-1-59745-558-9_6

[43] Bennett B.D., Kimball E.H., Gao M., Osterhout R., Van Dien S.J., Rabinowitz J.D. Absolute metabolite concentrations and implied enzyme active site occupancy in Escherichia coli. Nat. Chem. Biol. 2009; 5:593–599. 19561621 10.1038/nchembio.186 PMC2754216

[44] Nicholson D., Sengupta A., Sung H.-L., Nesbitt D.J. Amino acid stabilization of nucleic acid secondary structure: insights from single molecule studies. J. Phys. Chem. B. 2018; 122:9869–9876. 30289262 10.1021/acs.jpcb.8b06872 PMC6224135

[45] Gundlach J., Herzberg C., Hertel D., Thumer A., Daniel R., Link H., Stulke J. Adaptation of bacillus subtilis to life at extreme potassium limitation. MBio. 2017; 8:e00861–17. 28679749 10.1128/mBio.00861-17 PMC5573677

[46] Epstein W. The roles and regulation of potassium in bacteria. Progress in Nucleic Acid Research and Molecular Biology. 2003; 75:293–320. 14604015 10.1016/s0079-6603(03)75008-9

[47] Savinov A., Perez C.F., Block S.M. Single-molecule studies of riboswitch folding. Biochim. Biophys. Acta - Gene Regul. Mech. 2014; 1839:1030–1045. 10.1016/j.bbagrm.2014.04.005 PMC4177941 24727093

[48] Ray S., Chauvier A., Walter N.G., Chauvier A., Walter N.G. Kinetics coming into focus: single-molecule microscopy of riboswitch dynamics. RNA Biol. 2018; 00:1–9. 10.1080/15476286.2018.1536594 PMC6693532 30328748

[49] McCluskey K., Carlos Penedo J. An integrated perspective on RNA aptamer ligand-recognition models: clearing muddy waters. Phys. Chem. Chem. Phys. 2017; 19:6921–6932. 28225108 10.1039/c6cp08798a

[50] Wood S., Ferré-D’Amaré A.R., Rueda D. Allosteric tertiary interactions preorganize the c-di-GMP riboswitch and accelerate ligand binding. ACS Chem. Biol. 2012; 7:920–927. 22380737 10.1021/cb300014u PMC3356476

[51] Furtig B., Nozinovic S., Reining A., Fu B., Schwalbe H. Multiple conformational states of riboswitches fine-tune gene regulation. Curr. Opin. Struct. Biol. 2015; 30:112–124. 25727496 10.1016/j.sbi.2015.02.007

[52] Lemay J.-F., Penedo J.C., Tremblay R., Lilley D.M.J., Lafontaine D.A. Folding of the Adenine Riboswitch. Chem. Biol. 2006; 13:857–868. 16931335 10.1016/j.chembiol.2006.06.010

[53] Dalgarno P.A., Bordello J., Morris R., St-Pierre P., Dubé A., Samuel I.D.W., Lafontaine D.A., Carlos Penedo J. Single-molecule chemical denaturation of riboswitches. Nucleic Acids Res. 2013; 41:4253–4265. 23446276 10.1093/nar/gkt128 PMC3627600

[54] Brenner M.D., Scanlan M.S., Nahas M.K., Ha T., Silverman S.K. Multivector fluorescence analysis of the xpt guanine riboswitch aptamer domain and the conformational role of guanine. Biochemistry. 2010; 49:1596–1605. 20108980 10.1021/bi9019912 PMC2854158

[55] Heppell B., Blouin S., Dussault A., Mulhbacher J., Ennifar E., Penedo J.C., Lafontaine D.A. Molecular insights into the ligand-controlled organization of the SAM-I riboswitch. Nat. Chem. Biol. 2011; 7:384–392. 21532599 10.1038/nchembio.563

[56] Suddala K.C., Wang J., Hou Q., Walter N.G. Mg2+ Shifts Ligand-Mediated Folding of a Riboswitch from Induced- Fit to Conformational Selection. J. Am. Chem. Soc. 2015; 137:14075–14083. 26471732 10.1021/jacs.5b09740 PMC5098500

[57] Haller A., Rieder U., Aigner M., Blanchard S.C., Micura R. Conformational capture of the SAM-II riboswitch. Nat. Chem. Biol. 2011; 7:393–400. 21532598 10.1038/nchembio.562

[58] Haller A., Altman R.B., Soulière M.F., Blanchard S.C., Micura R. Folding and ligand recognition of the TPP riboswitch aptamer at single-molecule resolution. Proc. Natl. Acad. Sci. U.S.A. 2013; 110:4188–4193. 23440214 10.1073/pnas.1218062110 PMC3600479

[59] Holmstrom E.D., Polaski J.T., Batey R.T., Nesbitt D.J. Single-molecule conformational dynamics of a biologically functional hydroxocobalamin riboswitch. J. Am. Chem. Soc. 2014; 136:16832–16843. 25325398 10.1021/ja5076184 PMC4277777

[60] Sung H., Nesbitt D.J. Single-molecule FRET kinetics of the Mn2+ riboswitch: evidence for allosteric Mg2+ control of “induced-fit” vs “conformational selection” folding pathways. J. Phys. Chem. B. 2019; 123:2005–2015. 30739441 10.1021/acs.jpcb.8b11841

[61] Reining A., Nozinovic S., Schlepckow K., Buhr F., Fürtig B., Schwalbe H. Three-state mechanism couples ligand and temperature sensing in riboswitches. Nature. 2013; 499:355–359. 23842498 10.1038/nature12378

[62] Warhaut S., Mertinkus K.R., Philipp H., Boris F., Heilemann M., Hengesbach M., Schwalbe H. Ligand-modulated folding of the full-length adenine riboswitch probed by NMR and single-molecule FRET. Nucleic Acids Res. 2017; 45:5512–5522. 28204648 10.1093/nar/gkx110 PMC5605240

[63] Eschbach S., St-Pierre P., Penedo J.C., Lafontaine D.A. Folding of the SAM-I riboswitch: a tale with a twist. RNA Biol. 2012; 9:535–541. 22336759 10.4161/rna.19648

[64] Roy S., Hennelly S.P., Lammert H., Onuchic J.N., Sanbonmatsu K.Y. Magnesium controls aptamer-expression platform switching in the SAM-I riboswitch. Nucleic Acids Res. 2019; 47:3158–3170. 30605518 10.1093/nar/gky1311 PMC6451092

