# Supplementary material for: Unprecedented tunability of riboswitch structure and regulatory function by sub-millimolar variations in physiological Mg2+
Source: Nucleic Acids Res. 2019 May 2;47(12):6478–87. doi: 10.1093/nar/gkz316 (PMC6614840; doi:10.1093/nar/gkz316)
Supplement: gkz316_Supplemental_File [file gkz316_supplemental_file.docx]

**Unprecedented tunability of riboswitch structure and regulatory function by sub-millimolar variations in physiological Mg^2+^**

Kaley McCluskey^1,2*^, Julien Boudreault^3^, Patrick St-Pierre^3^, Cibran Perez-Gonzalez^1,4^, Adrien Chauvier^3^, Adrien Rizzi^5^, Pascale B. Beauregard^6^, Daniel A. Lafontaine^3*^, and J. Carlos Penedo^1,7*^

^1^SUPA School of Physics and Astronomy, University of St. Andrews, Scotland, UK KY16 9SS

^2^Present address: Department of Bionanoscience, Delft University of Technology, Delft, The Netherlands, 2629HZ

^3^Département de Biologie, Université de Sherbrooke, Québec, Canada J1K 2R1

^4^Present address: Centro Singular the Investigation en Quimica Biologica y Materiales Moleculares, Universidad de Santiago de Compostela, Santiago de Compostela, Spain, 15782

^5^Département de Chimie, Faculté des Sciences, Université de Sherbrooke, Sherbrooke, Canada

^6^Centre SÈVE, Département de Biologie, Faculté des Sciences, Université de Sherbrooke, Sherbrooke, Canada

^7^Biomedical Sciences Research Complex, School of Biology, University of St. Andrews, Scotland, UK KY16 9ST

*To whom correspondence should be addressed

**SUPPLEMENTARY METHODS**

**Construction of the *lysC* aptamer and variants**

The lysine aptamer was generated using a three-strand hybridization approach as shown in Figure 1. The oligonucleotide sequences used to construct the wild type and each of the variants and the position of each dye and the biotin group are summarized in Table 1. The longest strand (LMS, Table 1) was obtained using *in vitro* transcription and comprises the 5’ end of P1, P2-P4 and an extended P5. A 5’ biotinylated DNA tether (DNA-bi, Table 1) that binds the P5 extension was obtained by solid-phase synthesis (IDT Inc., USA) and used to immobilize the aptamer to the surface of the quartz microscope slide. The third strand (1-5V2, Table 1) was obtained by solid-phase synthesis incorporating the Cy3 donor at the 5’ end and an internal amino modifier for post-synthetic attachment of the Cy5 acceptor. Both P1 and P5 have been extended by 5 bp to stabilize the formation of the LMS and 1-5V2 duplex regions.

**Molecular modelling of dye positions**

The three-dimensional FRET model uses the crystallographic structure of the ligand-bound lysine aptamer from *Thermotoga maritima* (PDB: 3DIL) (1) as the template, to which elongated stems P1 and P5 are added with the sequences of the experimental constructs. Importantly, the modelled construct incorporates part of the tether sequence for a more accurate representation of the labelled aptamer. Stems P1 and P5 were built following an RNA A-form helix with the use of the UCSF Chimera software (2). The modelled stems incorporate two extra base pairs that are overlapped with their counterparts in the crystal structure using Pymol (The PyMOL Molecular Graphics System, Version 1.7.4 Schrödinger, LLC) to achieve a correct alignment. The modelling of the dyes was done using the FRET Positioning and Screening Software tool available from the Seidel lab webpage (3). The software approximates each dye to a sphere defined by three different radii that is attached to the DNA via a flexible linker defined by its length (L_linker_) and width (w_linker_) (For Cy3: L_linker_=20 Å, w_linker_=4.5 Å, R_dye1_=6.8 Å, R_dye2_=3.0 Å, R_dye3_=1.5 Å. For Cy5: L_linker_=22 Å, w_linker_=4.5 Å, R_dye1_=11 Å, R_dye2_=3.0 Å, R_dye3_=1.5 Å). The software uses these parameters to calculate the dye accessible volume (AV), which simulates all the accessible positions through bending of the linker from the attachment point(Kalinin *et al.*, 2012). The attachment positions used for these calculations correspond to those of the experimental constructs (Table 1). The modelled dyes can be then used to provide the mean and FRET-averaged distances, with the latter resulting from the integration of all possible FRET efficiencies. These calculations report a mean distance of 53.6 Å.

**RNA transcription and purification**

Strand LMS was transcribed from a double stranded DNA template using T7 RNA polymerase (Thermo Scientific). Templates were generated by recursive PCR from purified synthetic oligonucleotides. RNA was purified by denaturing 10% polyacrylamide gels containing 7 M urea, electroeluted into 8 M ammonium acetate and recovered by ethanol precipitation

**Post-synthetic dye labelling and aptamer annealing**

The 1-5V2 strand carrying an amino modifier at the indicated position was labelled using a succinimidyl ester derivative of the Cy5 acceptor dye according to the manufacturer’s protocol (GE Life Sciences, USA). RNA strands were purified using denaturing PAGE as above and annealed by heating a mixture of the three strands (1:1 molar ratio) to 90° C in 10 mM Tris buffer (pH 7.5), 50 mM NaCl, and slowly cooling to room temperature. Full-length hybridized aptamers were purified by native PAGE and ethanol precipitated.

**Single-molecule analysis of FRET distributions and kinetic rates**

Single-molecule FRET histograms obtained as a function of Mg^2+^ or lysine concentration were obtained by averaging the first 10 frames of each FRET trace for every individual molecule after filtering photobleaching and blinking effects. Idealized single-molecule trajectories were obtained using Hidden Markov Modelling implemented in a software package available from the Single-Molecule Nanometry Group at the University of Illinois at Urbana-Champaign (USA)(4). TDP plots were generated using a custom-made software package available from the same group. States were identified by fitting the single-molecule histogram to a sum of Gaussian functions, and the centre of each individual Gaussian was assigned as the average FRET value for each specific state. For distributions containing more than one FRET state, the relative population of each state was determined from the area under the curve describing that particular state normalized with respect to the total area of all the states present.

Single-molecule FRET isotherms as a function of Mg^2+^ were fitted to the Hill equation, shown below (Eq. 1). A similar expression was applied in the case of isotherms obtained as a function of lysine ligand by replacing the Mg^2+^ concentration by the concentration of ligand.

| $E_{app}=E_{o}+\left( \Delta E \right)\left[ \frac{\left( {Mg}^{2+} \right)^{n}}{K_{D}^{n}+\left( {Mg}^{2+} \right)^{n}} \right]$ | Eq. 1 |
| --- | --- |

Here, E_o_ represents the FRET efficiency in the absence of metabolite, ∆E indicates the total change in FRET efficiency during the titration, K_D_ represents the dissociation constant, and n is the Hill coefficient.

Single-molecule dwell-time histograms were fitted to mono-exponential functions to extract the kinetic rate for each specific transition. Because rapidly fluctuating molecules undergo more transitions that slowly fluctuating ones, to avoid biasing towards fast rates, dwell time histograms were obtained using a weighting factor inversely proportional to the number of transitions observed for each molecule. The variation of the observed transition rate as a function of lysine ligand concentration was fitted to Eq. 2 shown below:

| $k_{obs}=\left( k_{fold} \right)\left[ \frac{\left( Lysine \right)^{n}}{K_{D, bind}^{n}+\left( Lysine \right)^{n}} \right]$ | Eq. 2 |
| --- | --- |

Here, k_obs_ indicates the observed rate for a specific FRET transition, k_fold_ is the intrinsic folding rate, K_D_ is the ligand dissociation constant, and n is the Hill coefficient that for a 1:1 complex stoichiometry has been fixed to 1.

**References**

1. Serganov, A., Huang, L. & Patel, D. J. Structural insights into amino acid binding and gene control by lysine riboswitch. *Nature* **455**, 1263-1268 (2008).
2. Pettersen, E.F., Goddard, T.D., Huang, C.C., Couch, G.S., Greenblatt, D.M., Meng, E.C., Ferrin, T.E. UCSF Chimera--a visualization system for exploratory research and analysis. J. Comput. Chem. **13**, 1605–12 (2004).
3. Kalinin, S. *et al.* A toolkit and benchmark study for FRET-restrained high-precision structural modeling. *Nature Methods* **9**, 1218-1225 (2012).
4. McKinney, S. A., Joo, C. & Ha, T. Analysis of single-molecule FRET trajectories using hidden Markov modeling. *Biophysical journal* **91**, 1941-1951 (2006).

**SUPPLEMENTARY FIGURES AND TABLES**

| 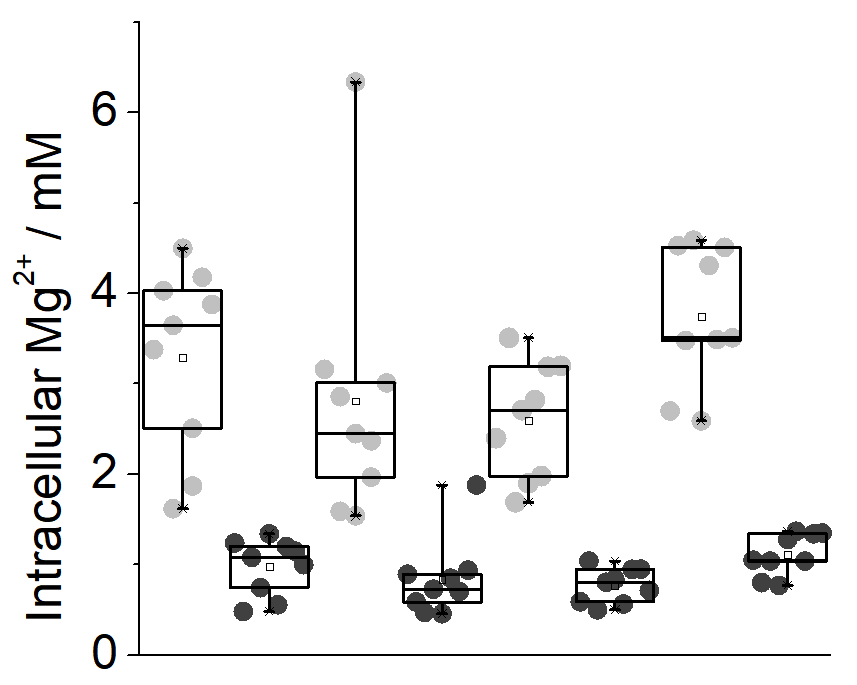 1h  2h  3h  4h  **Upper limit**  **Lower limit** |
| --- |
| Figure S1. Box plot showing the distribution of maximum (light grey) and minimum (dark grey) values of intracellular free Mg^2+^ concentration *in vivo* in *B. Subtilis* during its growth phase, obtained using inductively-coupled plasma mass spectrometry (ICP-MS). The lower and upper boundaries of the box are the first and third quartiles, respectively. The whiskers (vertical lines) indicate the absolute minimum and maximum values. |
| 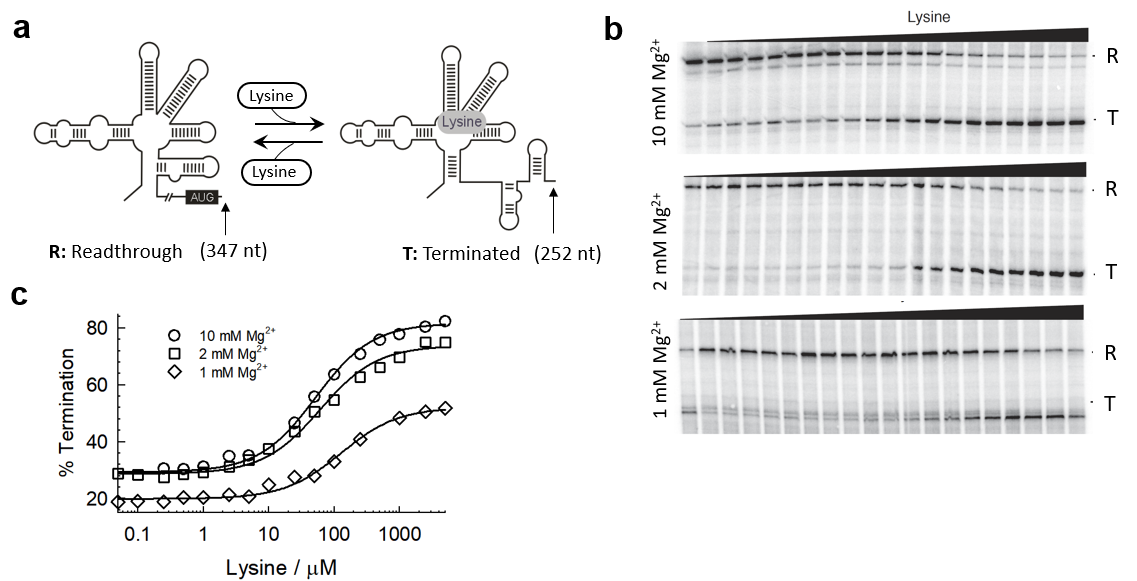 |
| **Figure S2. The function of lysC riboswitches is tightly controlled by the concentration of Mg^2+^ ions.** (a) Single-round *in vitro* transcription assays performed on the wild-type *lysC* riboswitch as a function of lysine concentration. In the absence of ligand, the RNAP transcribes the complete 347 mRNA sequence (**R**, readthrough product). In contrast, ligand binding to the aptamer allows the terminator stem to be formed, leading to premature transcription termination (**T**, 252 nt terminated product). (b) Single-round transcriptions performed in the presence of increasing concentrations of lysine at 10 mM (upper panel), 2 mM (middle panel) and 1 mM (bottom panel) Mg^2+^ resolved on 5% denaturing polyacrylamide gels in a background of 100 mM K^+^. Readthrough and terminated products are indicated on the right. (c) Percentage of termination as a function of lysine concentration at each concentration of Mg^2+^ ions. The solid line represents the fit to a Hill model (Supplementary Methods). T_50_ values of 52 ± 7 μM, 61 ± 7 μM and 129 ± 15 μM were obtained at 10 mM, 2 mM and 1 mM Mg^2+^, respectively. |
| 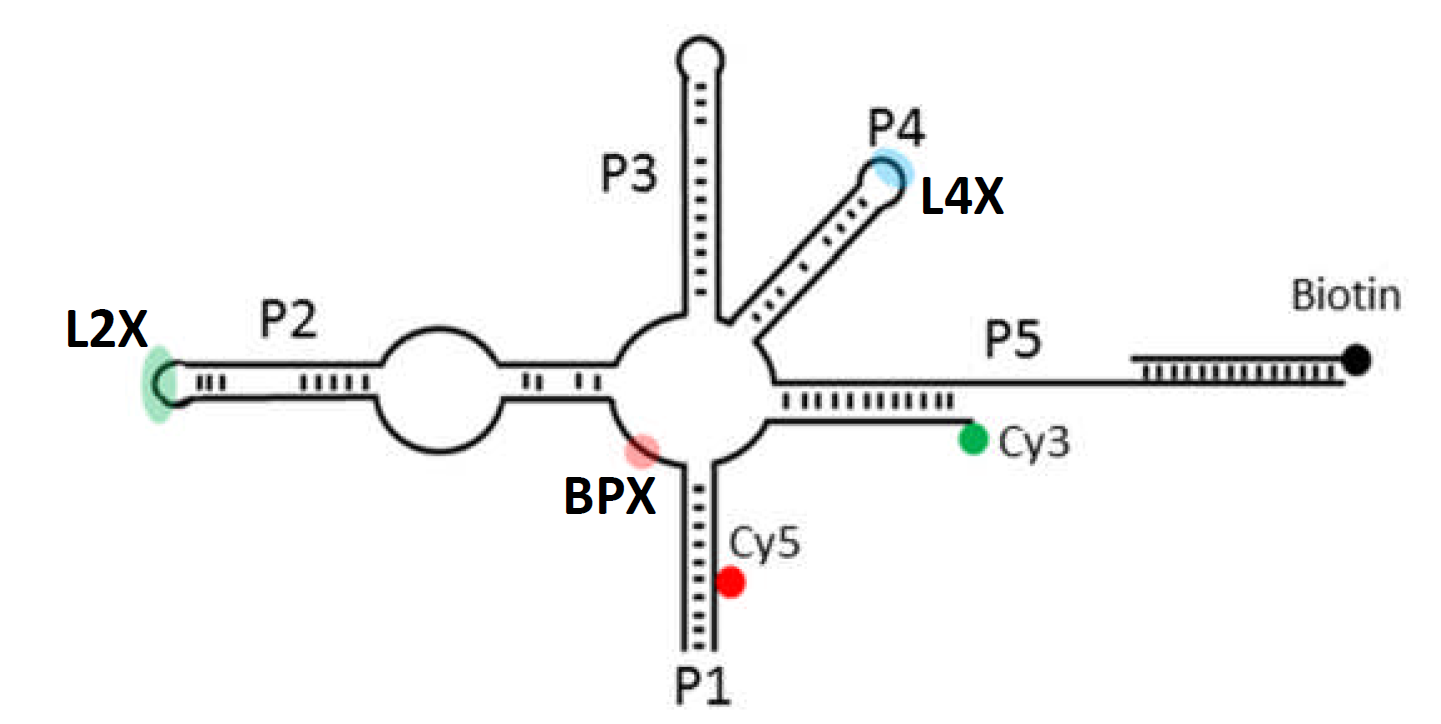 |
| **Figure S3.** Experimental construct for TIR-FRET experiments on the lysine riboswitch**.** P1 and P5 are extended from 6 and 7 bp to 11 and 12 bp, respectively. Cy3 and Cy5 are attached to a separate RNA strand that is annealed to the rest of the riboswitch to form the full construct. P5 has a single-stranded extension that hybridizes to a single DNA strand carrying a biotin group. |

| 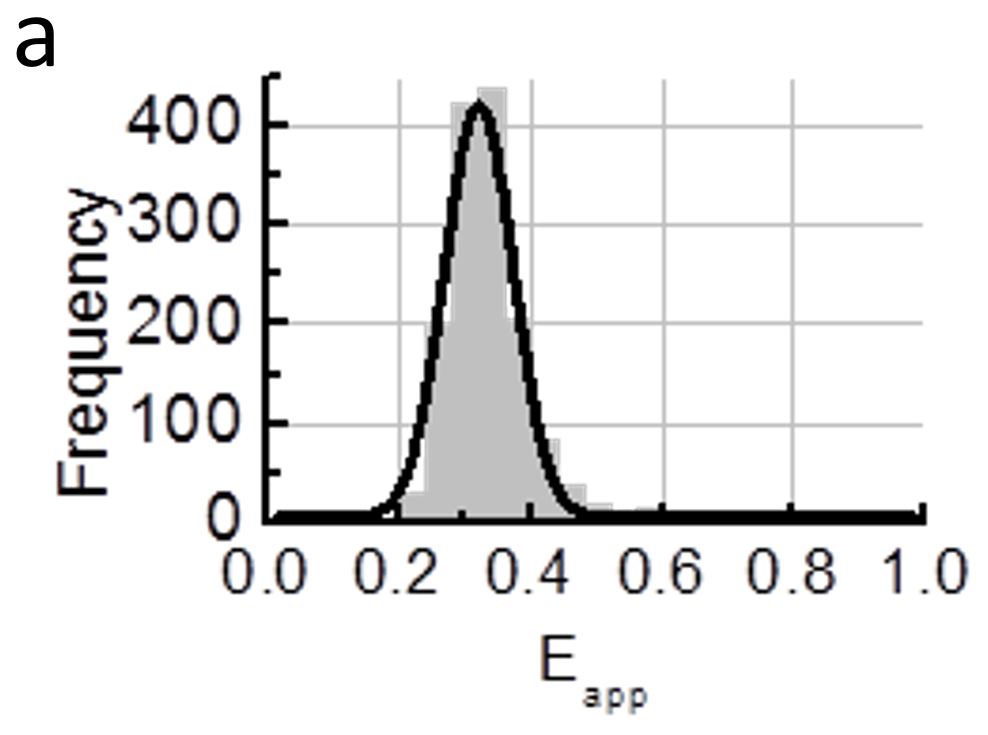 | 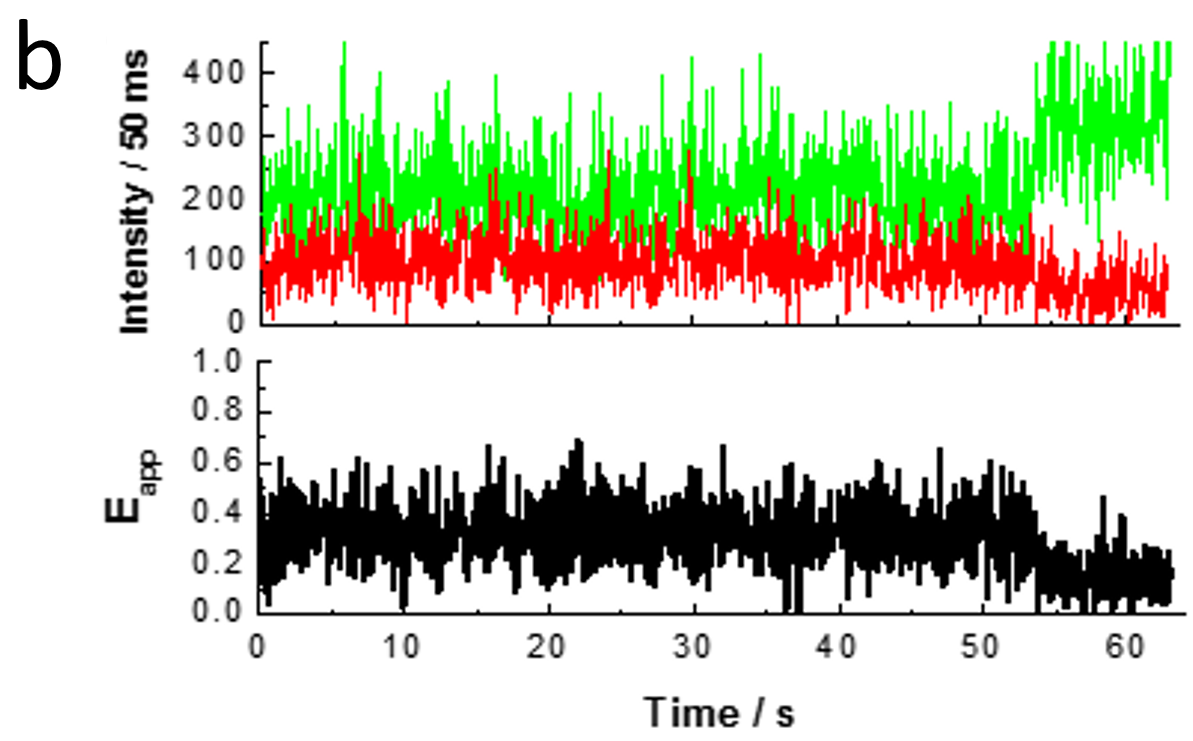 |
| --- | --- |
| **Figure S4.** Population histogram (a) and representative trajectory (b) in 50 mM NaCl. | |

| 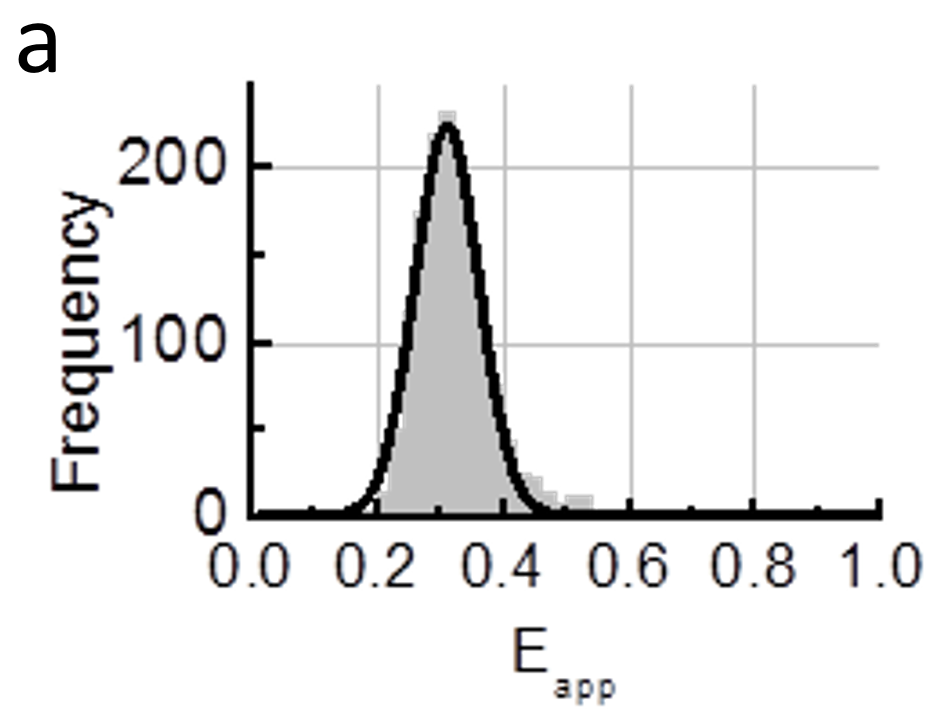 | 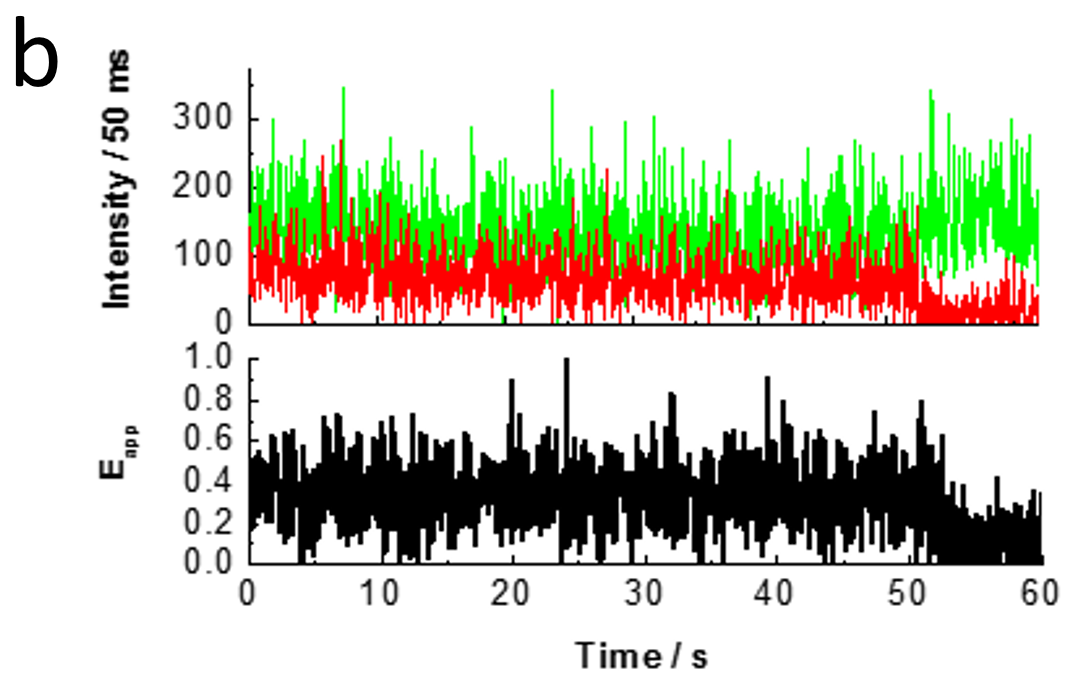 |
| --- | --- |
| **Figure S5.** Population distribution (a) and representative single-molecule trace (b) for the lysine aptamer in 2 mM EDTA. The population is monomodal in the unfolded state, **U**, centered at E_FRET_ = 0.31 ± 0.01. Donor, acceptor and FRET trajectories are shown in green, red and black, respectively. | |

| 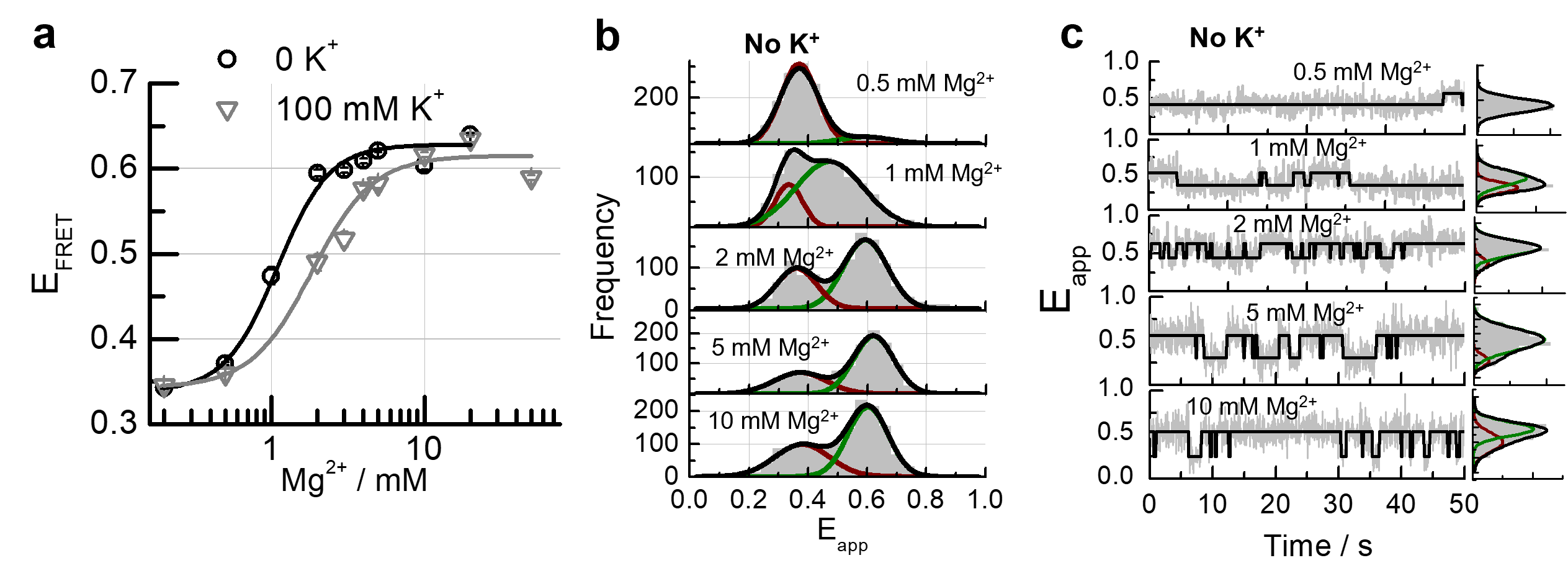 |
| --- |
| **Figure S6.** (a) Variation in FRET efficiency as a function of Mg^2+^ ions in the absence (ο) and presence (∆) of 100 mM K^+^ . The FRET efficiency at each Mg^2+^ concentration was obtained from the average of the FRET efficiency of more than 1000 molecules. The solid lines represent the fit to a Hill model (see Supplementary Methods for details). (b) Single-molecule FRET histograms obtained at the indicated concentrations of Mg^2+^ in the absence of K^+^. The solid black line represents the fit to two Gaussian populations, and the red and green lines indicate the relative contributions of the unfolded (U) and ligand-free folded states (F_LF_), respectively. (c) Representative single-molecule FRET trajectories showing the frequency of fluctuations between the U and F_LF_ states as a function of Mg^2+^ concentration. For each trajectory, the single-molecule FRET histogram and the corresponding fit to a two-Gaussian function are also shown (right panels). The solid line represents the idealized FRET trajectory obtained using a Hidden Markov Modelling approach (see Supplementary Methods for details). |

| 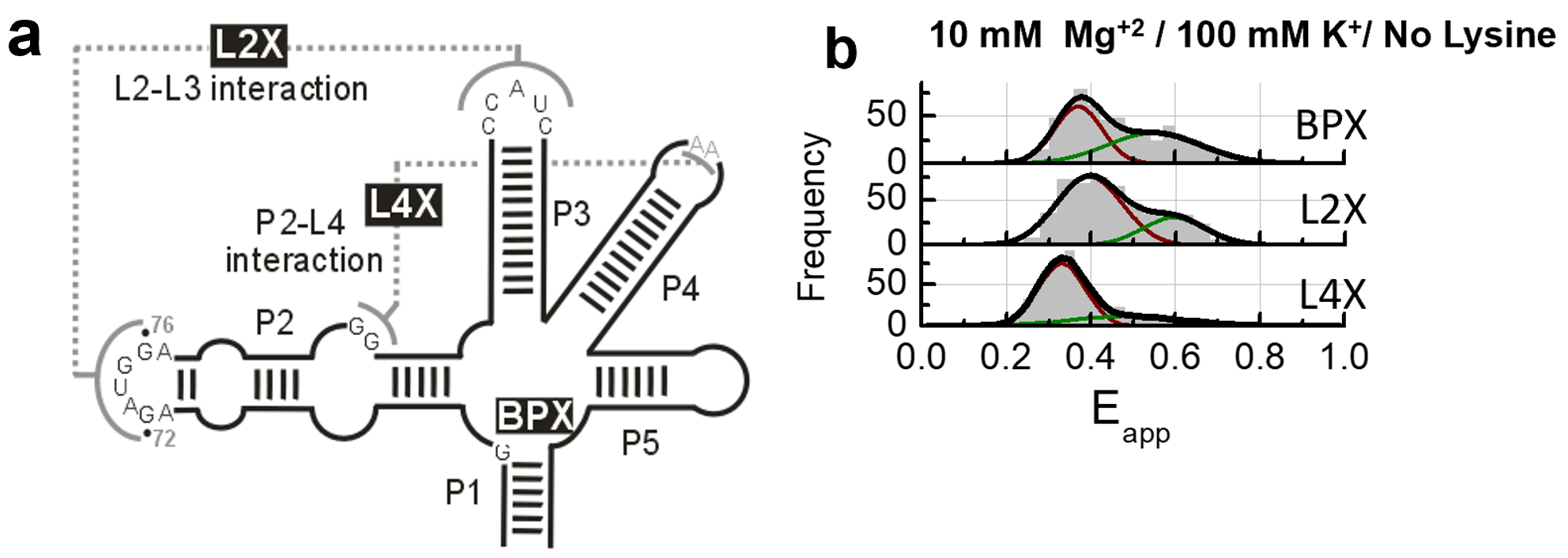 |
| --- |
| **Figure S7.** (a) Schematics of the three aptamer variants used in this study to determine the range of tertiary contacts formed in the ligand-free folded state. The L2X and L4X variants disrupt the formation of the L2-L3 and P2-L4 interactions, respectively, whereas the BPX variants is designed to avoid ligand binding. L2X: L2-L3 knockout in which nucleotides G72-G76 were changed to CUACC. L4X: P2-L4 knockout in which nucleotides A156/157 were changed from AA to CC. BPX: nucleotide G39 was changed to C (see Table S1). (b) Single-molecule FRET histograms obtained for each variant in the absence of ligand and in a background of 100 mM K^+^ and 10 mM Mg^2+^ confirmed the unfolded state as the predominant structure for the three variants. The black solid line represents the fitting to a two Gaussian model and the red and green lines represent the contributions from the U and F_LF_ states. |

| 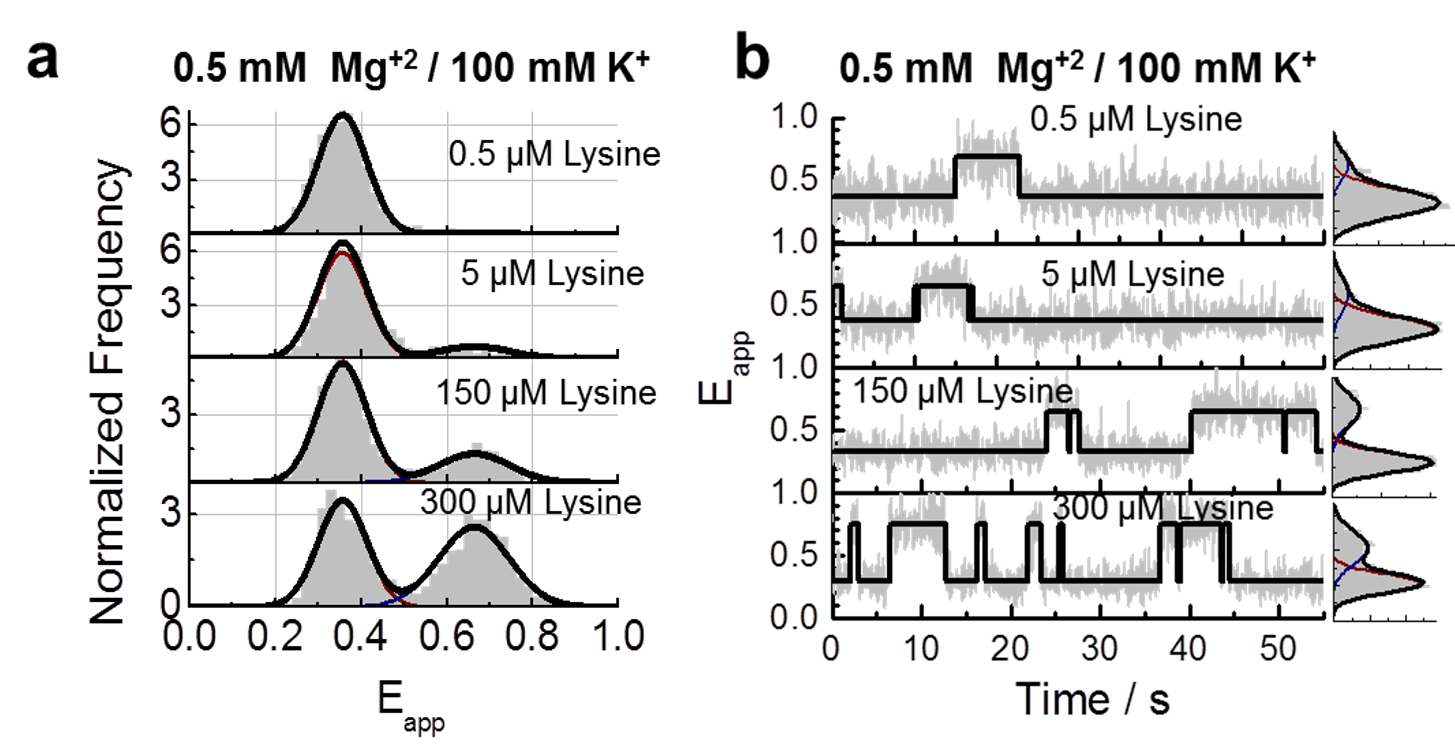 |
| --- |
| **Figure S8.** (a) Single-molecule FRET histograms obtained at the indicated concentrations of lysine in 0.5 mM Mg^2+^ and a background of 100 mM K^+^. Increasing the concentration of lysine ligand promotes the formation of a new state, **F_LB_**, with an E_app_ value of ~ 0.7. The solid black line represents the fit to two Gaussian functions, except in 0.5 μM lysine, where only the **U** state is present. The red and blue lines represent the relative contributions of the **U** and **F_LB_** states, respectively, at each lysine concentration. (b) Representative single-molecule FRET trajectories and corresponding single-molecule FRET histograms (right panels) obtained in the same conditions as in (a), showing lysine-dependent fluctuations between two FRET states with values of 0.37 (**U** state) and 0.7 (**F_LB_** state). The solid line represents the idealized FRET trajectory obtained using a Hidden Markov modelling strategy. |

| 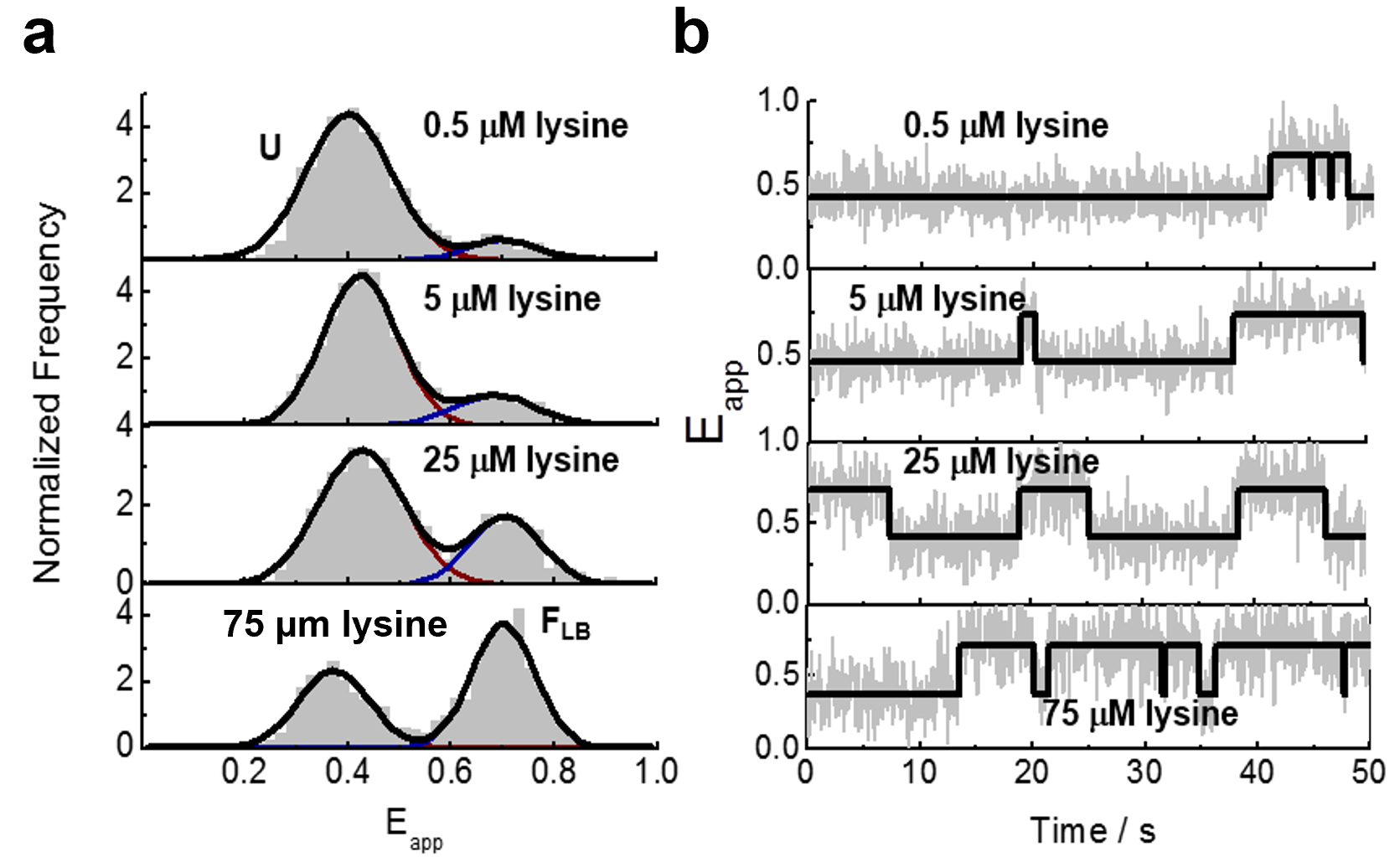 |
| --- |
| **Figure S9.** Single-molecule FRET histograms (a) and representative single-molecule FRET trajectories (b) obtained in a background of 1 mM Mg^2+^ and 100 mM K^+^ ions. Single-molecule FRET histograms have been fitted with two Gaussian populations (black lines). Individual Gaussians corresponding to the unfolded (**U**) and ligand-bound folded states (**F_LB_**) are shown in red and blue, respectively. Idealized single-molecule FRET trajectories extracted using Hidden Markov Modelling are shown as black lines. |

| 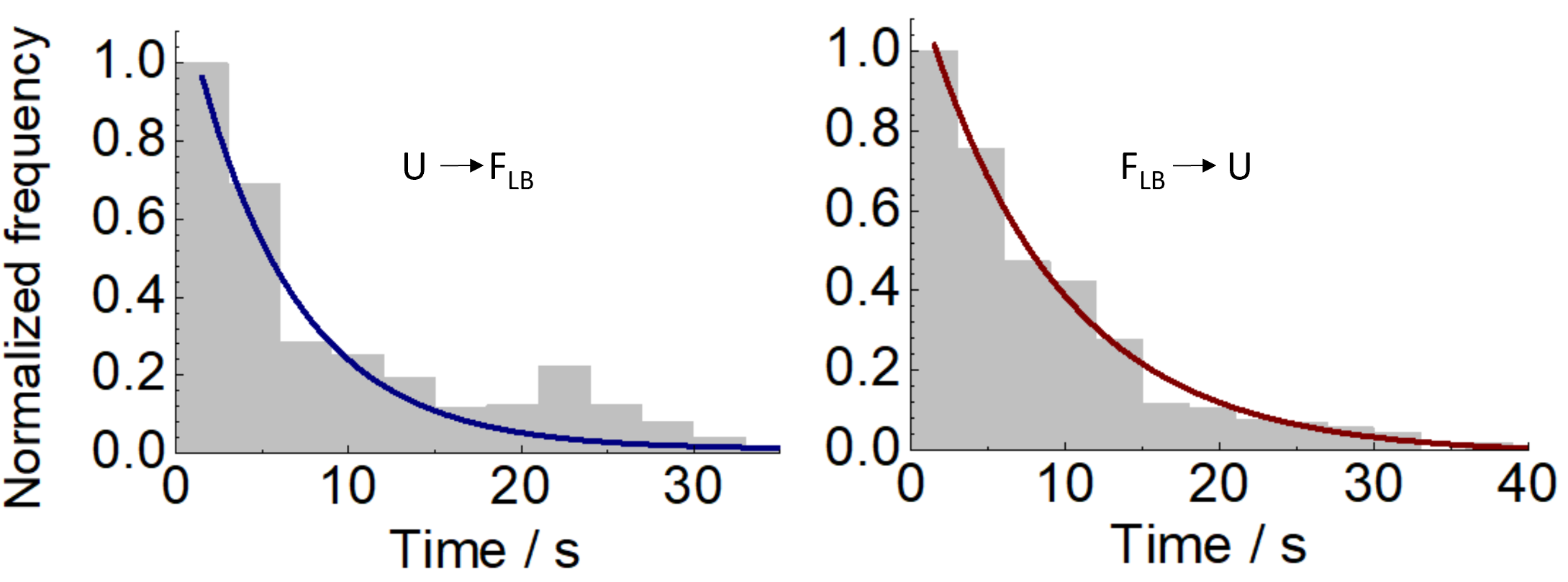 |
| --- |
| **Figure S10.** Representative dwell-time single-molecule histograms for transitions from **U** to **F_LB_** (left) and **F_LB_** to **U** (right) in 0.5 mM Mg^2+^ and 150 µM lysine. The blue and red lines are monoexponential fits with dwell times τ_U-Flb_ = 6.0 ± 0.9 s and τ_Flb-U_ = 8.9 ± 0.5 s. |

| 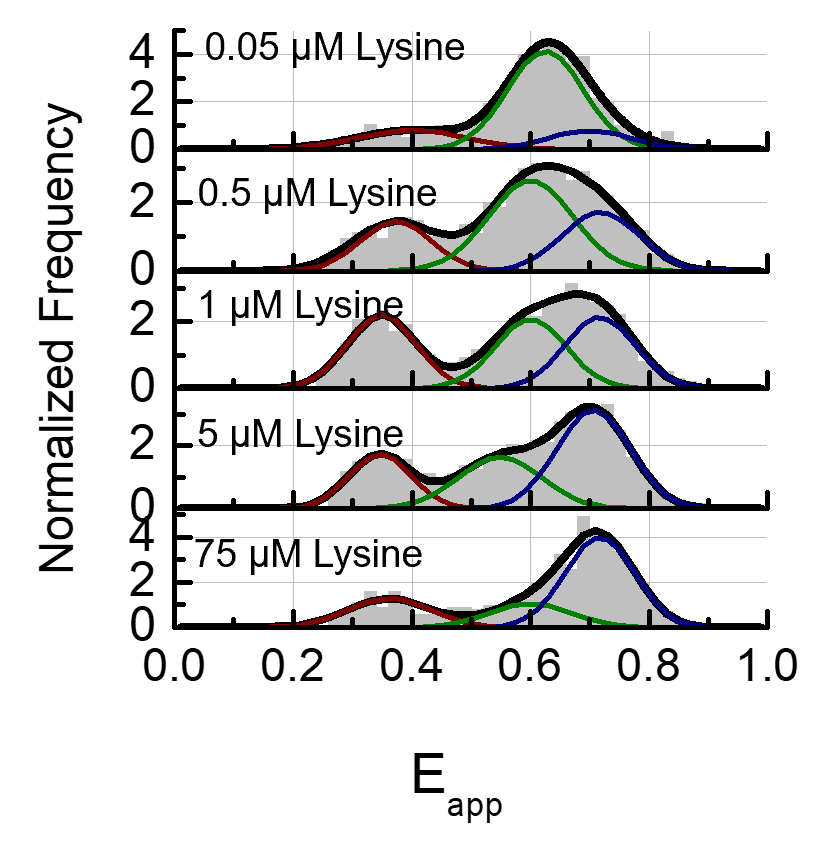 |
| --- |
| **Figure S11.** Single-molecule FRET histograms obtained at the indicated concentrations of lysine ligand in a background of 10 mM Mg^2+^ and 100 mM K^+^ ions. The solid black line represents the result from fitting each histogram to three Gaussians corresponding to the contribution of the **U** state (red), the folded ligand-free state (**F_LF_**) in green and the folded ligand-bound state (**F_LB_**) in blue. |

| 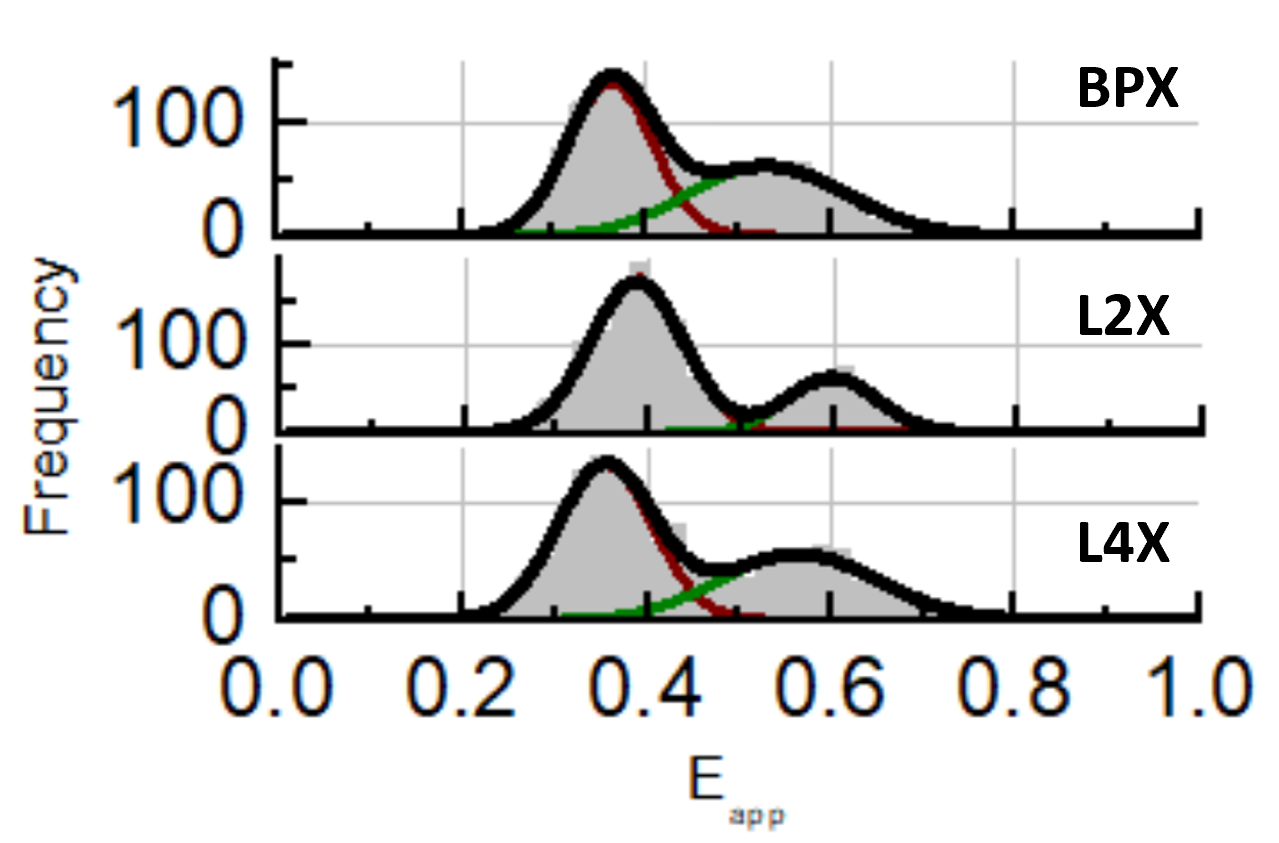 |
| --- |
| **Figure S12.** Single-molecule FRET histograms for the ligand binding incompetent aptamer variant (BPX) and the tertiary unfolded L2X and L4X variants, obtained in the presence of 2.5 µM lysine ligand in a background of 2 mM Mg^2+^ and 100 mM K^+^ ions |

| 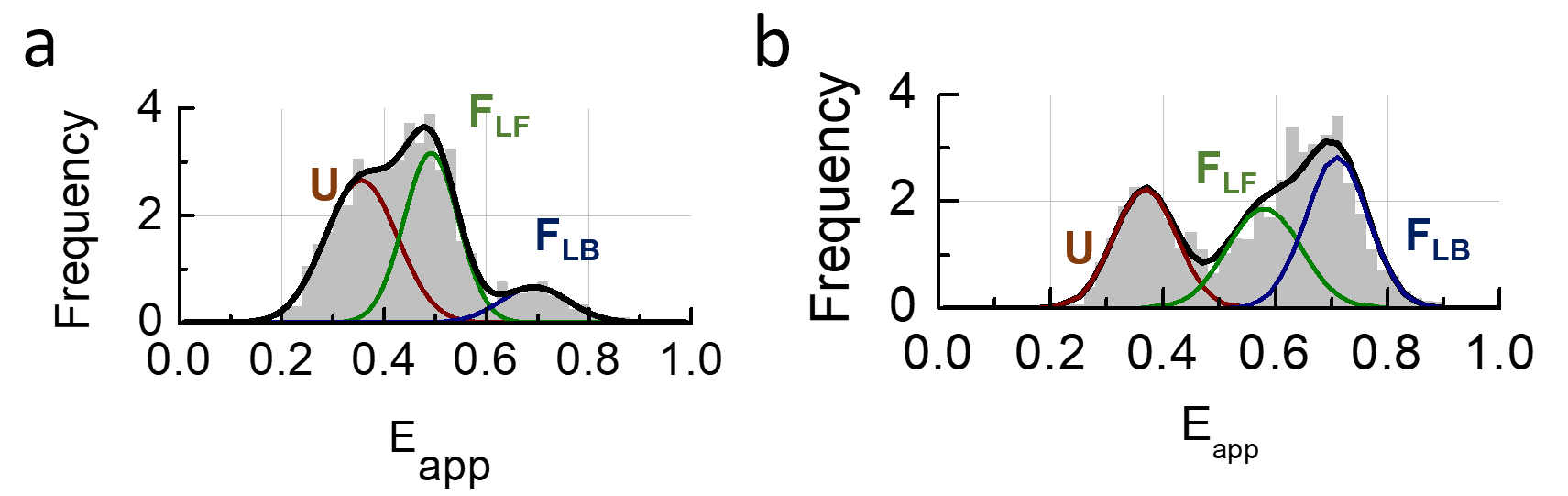 |
| --- |
| **Figure S13.** K^+^ ions are required to stabilize the folded ligand bound state and cannot be replaced by Na^+^ ions. (a) Single-molecule FRET histogram obtained in a background of 2 mM Mg^2+^ and 100 mM Na^+^ ions in the presence of 300 µM lysine ligand. (b) Single-molecule FRET histogram obtained in a background of 10 mM Mg^2+^ and 1 mM lysine without K^+^. The red line represents the fitting to three Gaussians and the red, green, and blue lines indicate the relative contributions of the unfolded (U), folded ligand-free (F_LF_) and folded ligand-bound states (F_LB_), respectively. |

| 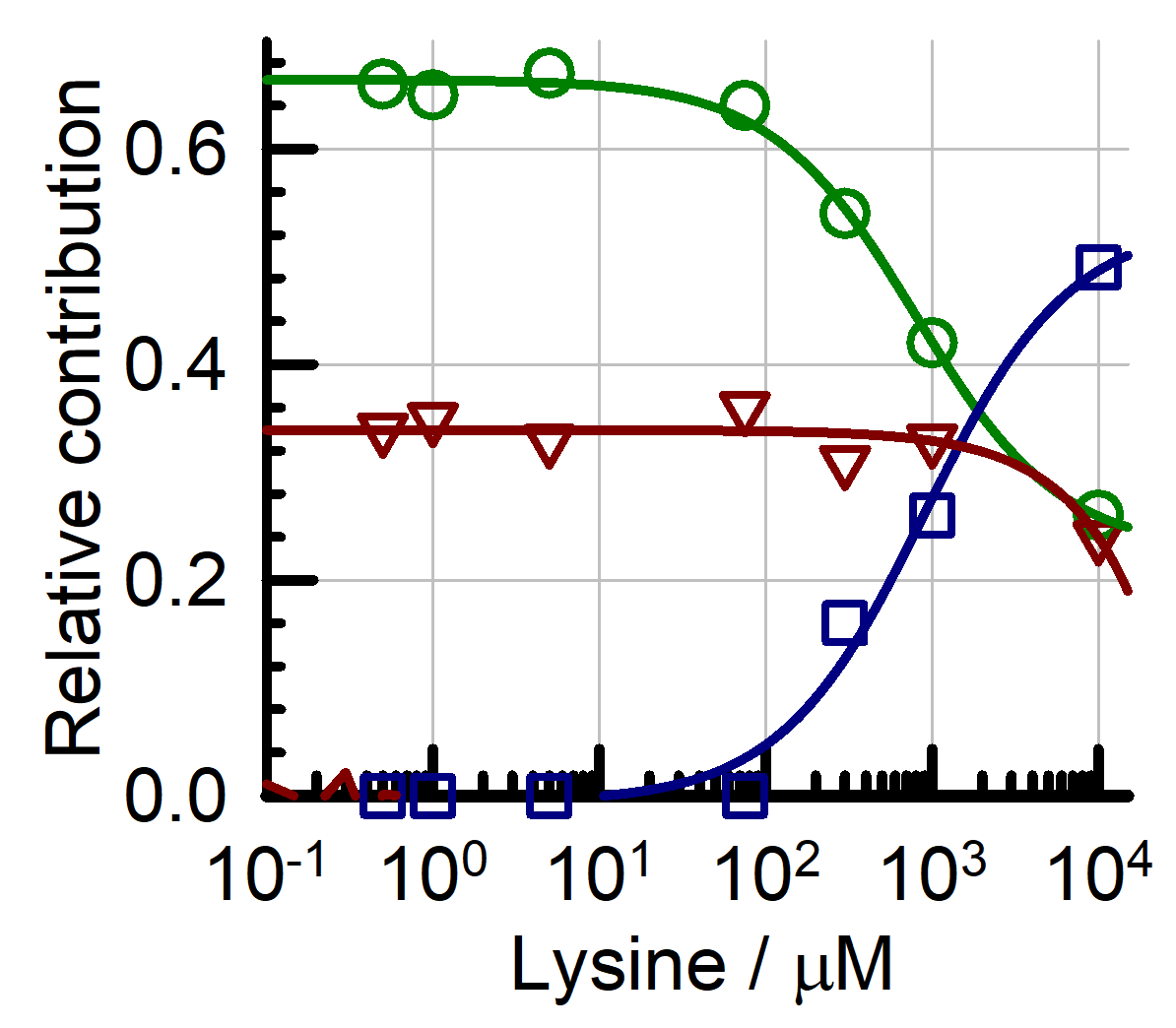 |
| --- |
| **Figure S14.** Relative contribution of the **U** (red), **F_LF_** (green) and **F_LB_** (blue) states as a function of lysine concentration in the absence of K^+^ ions. [Mg^2+^] = 10 mM. The solid lines represent the result of globally fitting the experimental data to a Hill model. The fitting provided values of 850 ± 35 µM and 1.1 ± 0.2 for the dissociation constant and Hill coefficient, respectively. |

**Table S1** Sequences of strands used to create the wild-type aptamer construct and its variants.

| Strand Name | Sequence | |
| --- | --- | --- |
| Main Strand (LMS) | 5’- | GCGGUGAAGAUAGA**G**GUGCGAACUUCAAGAGUAUGCCUUUGGAGAAA**GAUGG**AUUCUGUGAAAAAGGCUGAAAGGGGAGCGUCGCCGAAGCAAAUAAAACCCCAUCGGUAUUAUUUGCUGGCCGUGCAUUG**AA**UAAAUGUAAGGCUGUCAAGAAGCAUCGUUCAAGCGUUUUUUAUGCUUGGAAGAUAAGAA |
| BPX variant (G39C) |  | **G** → C |
| L2X variant (G72C-G76C) |  | **GAUGG** → CUACC |
| L4X variant (A156/157C) |  | **AA** → CC |
| P1-P5 dye strand (1-5V2) | 5’- | **Cy3**-CCGAUGCUUCUUGGAGGGCUAUCUU-**NCy5**-CACC |
| Tether (DNA-bi) | 5’- | **bi**-TTCTTATCTTCCAACGATAAAAAACGCTTG |

| **Table S2.** Dwell times (τ), kinetic rates, and corresponding free energy changes obtained for the U to F_LB_ transition as a function of lysine concentration in a background of 0.5 mM Mg^2+^. | | | | | |
| --- | --- | --- | --- | --- | --- |
| **Lysine / (µM)** | **τ_FLB_ / s** | **τ_U_ / s** | **k_U-FLB_ / s^-1^** | **k_FLB-U_ / s^-1^** | **ΔG / kcal mol^-1^** |
| **25** | 10 ± 2 | 23 ± 3 | 0.043 ± 0.006 | 0.10 ± 0.02 | 0.89 ± 0.02 |
| **75** | 5.7 ± 2 | 54 ±8 | 0.019 ± 0.003 | 0.18 ± 0.02 | 1.08 ± 0.02 |
| **150** | 8.9 ± 5 | 6.0 ± 9 | 0.17 ± 0.03 | 0.11 ± 0.01 | -0.12 ± 0.05 |
| **225** | 3.3 ± 3 | 3.4 ± 2 | 0.29 ± 0.02 | 0.30 ± 0.02 | 0.020 ± 0.001 |
| **300** | 4.3 ± 2 | 1.42 ± 6 | 0.70 ± 0.03 | 0.23 ± 0.01 | -0.65 ± 0.05  0 ± (5) |
| **1000** | 5.5 ± 4 | 0.82 ± 0.03 | 1.22 ± 0.04 | 0.18 ± 0.01 | -1.12 ± .01 |

| Table S3. Transition rates and energy landscape parameters for the lysine riboswitch’s ligand-binding transition at 0.5 mM Mg^2+^. For clarity, F_LF_ and F_LB_ are abbreviated to F and B, respectively, in the superscripts. | | | |
| --- | --- | --- | --- |
| Transition Rate (s^-1^ or s^-1^ M^-1^) or K_d_ (µM) | | Free Energy (kcal/mol) | |
| k_bind_^UB^ | (1.4±0.8) x 10^3^ | ΔG^0^_UB_(-L) | +0.89 ± 0.02 |
| k_fold_^UB^ | 1.3 ± 0.2 | ΔG^0^_UB_(+L) | -1.13 ± 0.01 |
| k_unfold_^BU^ | 0.18 ± 0.03 | ΔΔG^0^_UB_ | -2.0 ± 0.1 |
| K_UB,fold/bind_ | 310 ± 50 | ΔΔG^‡^_UB_ | -1.9 ± 0.1 |
|  |  | ΔΔG^‡^_BU_ | -0.34 ± 0.08 |

**Table S4.** Dwell times (τ), kinetic rates and sample sizes obtained for the interconversion dynamics of the lysine riboswitch in 2 mM Mg^2+^, 100 mM K^+^, and 2.5 µM lysine

|  | U→F_LF_ | F_LF_ →U | F_LF_ → F_LB_ | F_LB_ → F_LF_ | U→ F_LB_ | F_LB_ →U |
| --- | --- | --- | --- | --- | --- | --- |
| τ (s) | 0.83 ± 0.01 | 10 ± 2 | 74±16 | 28±2 | 0.44 ±0.05 | 4±1 |
| k (s^-1^) | 1.22 ± 0.01 | 0.10 ± 0.02 | 0.014 ± 0.003 | 0.036 ±0.003 | 2.3±0.3 | 0.25±0.06 |
| N | 443 | 343 | 151 | 170 | 41 | 36 |

Table S5. Dwell times (τ), kinetic rates, and corresponding free energy changes obtained for the F_LF_ to F_LB_ transition as a function of lysine concentration in a background of 2 mM Mg^2+^.

| Lysine / µM | τ_FLB_ / s | τ_FLF_ / s | k_FLF-FLB_ / s^-1^ | k_FLB-FLF_ / s^-1^ | ΔG / kcal mol^-1^ |
| --- | --- | --- | --- | --- | --- |
| **2.5** | 28 ± 2 | 74 ± 16 | 0.014 ± 0.003 | 0.035 ± 0.003 | 0.5 ± 0.1 |
| **5** | 28 ± 2 | 59 ± 8 | 0.017 ± 0.002 | 0.036 ± 0.002 | 0.43 ± 0.05 |
| **10** | 24 ± 1 | 28 ± 2 | 0.036 ± 0.002 | 0.041 ± 0.002 | 0.077 ± 0.005 |
| **25** | 26 ± 2 | 22 ± 2 | 0.044 ± 0.004 | 0.039 ± 0.003 | -0.072 ± 0.003 |
| **75** | 28 ± 2 | 6.9 ± 4 | 0.14 ± 0.01 | 0.035 ± 0.003 | -0.809 ± 0.007 |
| **150** | 31 ± 3 | 5.5 ± 2 | 0.18 ± 0.01 | 0.032 ± 0.003 | -1.01 ± 0.02 |
| **300** | 27 ± 2 | 2.8 ± 3 | 0.36 ± 0.04 | 0.037 ±0.003 | -1.33 ± 0.02 |

| Table S6. Transition rates and energy landscape parameters for the lysine riboswitch’s ligand-binding transition in 2 mM Mg^2+^. For clarity, F_LF_ and F_LB_ are abbreviated to F and B, respectively, in the superscripts. In the final two lines, the subscript B*u* indicates ΔΔG^‡^ between the barrier heights of the F_LB_ to F_LF_ and F_LB_ to U transitions. | | | |
| --- | --- | --- | --- |
| k_UF_ | 1.22 ± 0.01 | ΔG^0^_UF_ | -1.5 ± 0.1 |
| K_FU_ | 0.10 ± 0.02 |  |  |
| k_bind_^FB^ | (1.7 ± 0.1) x 10^3^ | ΔG^0^_FB_(-L) | +0.52 ± 0.07 |
| k_fold_^FB^ | 0.27 ± 0.08 | ΔG^0^_FB_(+L) | -1.33 ± 0.02 |
| k_unbind_^BF^ | 0.14 ± 0.06 | ΔΔG^0^_FB_ | -1.84 ± 0.05 |
| k_unfold_^BF^ | 0.037 ± 0.001 | ΔΔG^‡^_FB_ | -1.49 ± 0.09 |
| K_FB,fold_ | 0.14 ± 0.04 | ΔΔG^‡^_BF_ | +0.05 ± 0.01 |
| K_FB,bind_ | 80 ± 30 | ΔΔG^‡^_B_*_u_*(-L) | +0.61 ± 0.08 |
| K_FB,app_ | 11 ± 7 | ΔΔG^‡^_B_*_u_*(+L) | +1.01 ± 0.02 |
